# An Anthocyanin- and Anti-Ageing Amino Acids-Enriched Pigmented Rice Innovation Promotes Healthy Ageing Through the Modulation of Telomere, Oxidative Stress and Inflammation Reduction: A Randomized Clinical Trial

**DOI:** 10.3390/ijms262210911

**Published:** 2025-11-11

**Authors:** Jintanaporn Wattanathorn, Wipawee Thukham-mee, Sophida Phuthong, Weerapon Sangartit, Terdthai Thong-un, Praew Kotruchin, Thapanawong Mitsungnern, Suphap Im-uan, Nitiwat Sirijun, Supaporn Muchimapura

**Affiliations:** 1 Department of Physiology, Faculty of Medicine, Khon Kaen University, Mitraphap Road, Amphoe Muaeng, Khon Kaen 40002, Thailand; meewep@gmail.com (W.T.-m.); sophiph@kku.ac.th (S.P.); weerasan@kku.ac.th (W.S.); terdthai@kku.ac.th (T.T.-u.); nitisi@kku.ac.th (N.S.); supmuc@kku.ac.th (S.M.); 2 Research Institute for High Human Performance and Health Promotion, Khon Kaen University, Amphoe Muaeng, Khon Kaen 40002, Thailand; 3 Accident and Emergency Stroke Unit, Srinagarind Hospital, Faculty of Medicine, Khon Kaen University Khon Kaen, Khon Kaen 40002, Thailand; kpraew@kku.ac.th (P.K.); thapanawong@kku.ac.th (T.M.); supimo@kku.ac.th (S.I.-u.)

**Keywords:** anthocyanins, cognition, facial wrinkle, cardiovascular risk, telomere, oxidative stress, inflammation

## Abstract

Owing to the great demand for healthy ageing promotion, and the anti-ageing reputation of anthocyanins and amino acids, we aimed to assess the effect of anthocyanin- and anti-ageing amino acids-enriched pigmented rice innovation on age-related cognitive decline, facial wrinkles, and a cardiovascular risk, and explored its mechanisms and safety. A total of 90 male and female volunteers (45–65 years old) participated in a 3-arm randomized, double blinded, placebo-controlled parallel study for 12 weeks. They were randomly allocated to one of the following groups: placebo, “Zuper rice” (Zup) 2 g/day and “Zuper Rice” 4 g/day. Cognition, facial wrinkles, atherogenic index in plasma (AIP), telomere length, telomerase, oxidative stress and inflammatory markers, together with safety parameters, were assessed every 6 weeks until the end of the study and compared to the baseline data. A high dose of “Zup” improved cognition, facial wrinkles, AIP and oxidative stress, while a low dose of “Zup” improved cognition, telomere length, telomerase and inflammation. No toxicity signs were observed. Therefore, “Zup” is a potential healthy ageing promotion innovation which improves telomere length, telomerase activity and inflammation at a low dose, resulting in an improvement in cognitive decline and the suppression of oxidative stress. At a high dose, it gives rise to improvements in cognition, facial wrinkles and cardiovascular risk.

## 1. Introduction

The elderly population is estimated to be around 12% of the global population, and this number will triple by 2100 [1]. During the ageing process, both the physical and mental activities of this population group decrease due to an accumulation of molecular and cellular damage over time [2]. In addition, this period also increases vulnerability to numerous age-related disorders [3], which are chronic diseases and consume high healthcare expenditure [4]. Owing to the high impact of age-related disorders on the socio-economic burden, a preventive strategy against the aforementioned conditions, and healthy ageing promotion, have gained much attention.

“Healthy ageing” emphasizes a lifelong process of optimizing opportunities to improve and maintain a health-related quality of life (HQOL) [1]. It has been reported that the deterioration of body functions begins at middle age, which links youth and old age [5]. The preparation for healthy ageing should be commenced at the middle age period in order to allow a smooth transition into elderly life. This process often involves a proactive strategy to maintain or slow down the deterioration of physical and mental well-being. Among the various age-related functional changes, age-related cognitive decline has been regarded as one of the most important problems that affect a HQOL, because it can decrease self-care ability in daily life [6] and limit social activity engagement [7]. In addition, the visibility of skin ageing appears to be prominent during the middle age period. Recent studies have demonstrated a potential connection between the nervous system and skin, particularly facial skin, because they share the development from the neural crest, whereas the other parts of the skin develop from the mesoderm. Furthermore, a recent study also reveals that significant facial ageing is associated with cognitive impairment [8]. Facial skin ageing not only induces psychological distress [9], but also increases vulnerability to skin diseases, such as dermatitis [10], which in turn disturbs a HQOL. Based on the large impact of cognitive decline and skin ageing on a HQOL, and the association of the nervous system and facial skin mentioned earlier, a strategy that can protect against cognitive decline and facial skin ageing in the middle age population has gained attention.

Among various lifestyle interventions that play a crucial role in healthy life promotion is food [11]. Cumulative evidence demonstrates that consumption of antioxidants and anti-inflammation-enriched food can slow down cognitive decline and skin ageing [12]. These lines of evidence have revealed that anthocyanin-enriched substances possess antioxidant and anti-inflammatory effects [13,14]. They also improve cognition [15,16,17] and skin ageing [18,19]. Consumption of anthocyanin-enriched pigmented rice also exhibits antioxidant and anti-inflammatory effects in humans [20,21]. It also exhibits a neuroprotective effect [22,23], both in cell lines and in animal models. In addition, our previous work has demonstrated that pigmented rice, such as black sticky rice, also exhibits anti-stress, anti-anxiety and anti-depression in adult volunteers [24]. It also reveals anti-skin ageing by improving hydration and elasticity in human volunteers after daily application for 28 days [25]. Beyond anthocyanins, amino acids also exert anti-ageing effects, particularly anti-skin ageing [26,27] and anti-cognitive decline [28,29] effects.

Recently, it has been found that “Zuper Rice”, the rice (*Oryza sativa*) innovation developed from the mixture of three varieties of pigmented rice, Khao Kum, Hom Nil and Rice Berry, can improve both the texture and contents of the health beneficial ingredients, such as anthocyanins. It also contains a protein content around 9.89%, whereas the white rice that is commonly consumed, such as “Hom Mali” and “RD43”, showed a protein content around 6.94–8.55% [30]. In addition, it contains amino acids that promote cognitive enhancement, such as glycine [31], glutamate [32], arginine [33,34], leucine [35], aspartic acid [36] and glutamine [37], together with a cluster of amino acids such as “LP7” (consisting of leucine, phenylalanine, lysine, isoleucine, histidine, valine and tryptophan) [28,29]. Moreover, it also contains amino acids that are reputed to restore skin texture, such as branched chain amino acids (leucine, isoleucine and valine), arginine, glutamine and proline. The combination of branched chain amino acids (BCAA) and glutamine or proline can also restore dermal collagen protein synthesis impaired by ultraviolet (UV) irradiation [38]. Interestingly “Zuper rice” also contains amino acids which are reported to be associated with a reduction in cardiovascular risk, such as glutamine and alanine [39]. Owing to the high contents of anthocyanins and amino acids, reputed to have the cognitive enhancing, skin restoring and cardiovascular risk reducing effects mentioned earlier (the details of the amino acid contents of “Zuper rice” is provided in Supplementary Material File S1), together with “Zuper Rice’s” high content of protein that is required for the elderly [40] and its healthy ageing promotion effect by slowing down age-related changes such as cognitive decline, facial wrinkles and cardiovascular risk, “Zuper Rice” has gained much attention. Since most rice consumption by Asian people is in the form of cooking rice which has been exposed to a high temperature for a period of time, we focused on the effect of “Zuper rice” which was subjected to heat at the same temperature as that in the rice cooking process (90 °C, exposure time 30 min). According to this process, the content of anthocyanins in “Zuper rice” was approximately 1.731 ± 0.017 mg/g dry weight of “Zuper Rice”. We hypothesized that this pigmented rice innovation which is subjected to heat at the same temperature as the heat obtained from the cooking process can promote healthy ageing by slowing down cognitive decline and reducing facial wrinkles and cardiovascular risk in middle-aged volunteers. To the best of our knowledge, no clinical data regarding this aspect were available until now. Therefore, the purpose of this study was to prove this hypothesis and to explore the possible underlying mechanisms together with consumption safety.

## 2. Results

**Figure 1:**
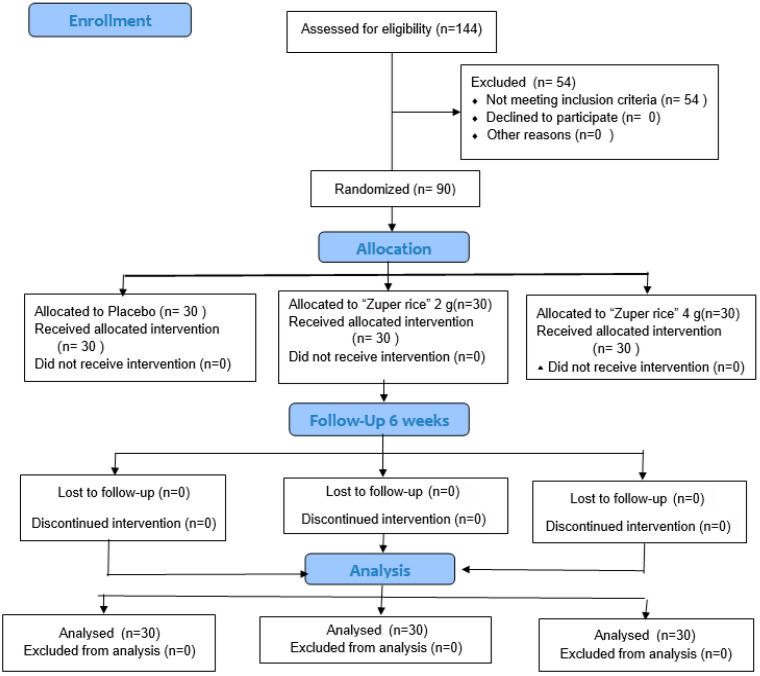
Consort diagram of subjects.

A total of 144 males and females aged between 45 and 65 years old were recruited to participate in this study. After screening for eligibility, only 90 subjects met the inclusion criteria. All subjects provided written informed consent. They were randomly divided into one of the following groups: placebo group (*n* = 30), “Zuper rice” 2 g/day (*n* = 30) and “Zuper rice” 4 g/day (*n* = 30). All subjects participated in the study throughout the study period. The flow diagram of subjects is shown in Figure 1.

The general characteristics of the subjects are shown in Table 1. It was found that there were no significant differences in the mean age, vital signs, weight, height or BMI of the subjects among these three groups at the baseline or before starting the intervention and throughout the 12-week study period. The distribution of male/female subjects in all treated groups also showed no difference.

### 2.1. Effect of “Zuper Rice” on Cognition

In this study, working memory was assessed and used as an indicator of cognitive function. Table 2 reveals that, after a 6-week intervention period, subjects who consumed “Zuper rice” at a dose of 2 g per day showed an improvement in % accuracy response of choice reaction time (*p*-value < 0.05, compared to the placebo group) whereas “Zuper rice” at a dose of 4 g per day significantly increased % accuracy response of digit vigilance (*p*-value < 0.05, compared to the placebo group). However, these changes disappeared during the 12 weeks of the consumption period. At this point, subjects who consumed “Zuper rice” at a dose of 2 g per day showed a significant response time reduction in the picture recognition test (*p*-value < 0.05, compared to the placebo group). No other parameters showed significant changes.

### 2.2. Effect of “Zuper Rice” on Facial Wrinkles

**Figure 2:**
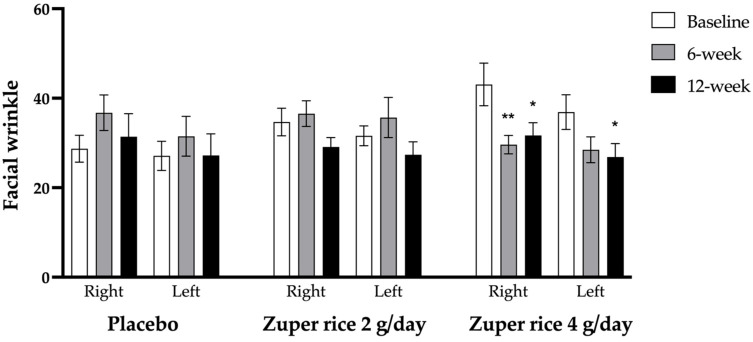
Facial wrinkles of subjects in the three groups: placebo, “Zuper rice” 2 g/day and “Zuper rice” 4 g/day (*n* = 30/gr). Data are expressed as mean ± SD. *, ** *p*-value < 0.05 and 0.01, respectively, compared to the baseline of each group.

Figure 2 reveals that, before the intervention, no significant differences in any investigated parameters among the three groups were observed. After 6 weeks of intervention, subjects who consumed “Zuper rice” at a dose of 4 g per day revealed a significant reduction in facial wrinkles. Subjects who consumed “Zuper rice” at a dose of 4 g/day revealed significantly decreased facial wrinkles on the right side at a 6-week intervention period (*p*-value < 0.01, compared to the baseline data), while the placebo-treated group failed to show a significant improvement in wrinkles. When the intervention period was prolonged to 12 weeks, a significant improvement in the wrinkles on both sides (*p*-value < 0.05 all, compared to the baseline data) was observed in subjects who consumed this dose of “Zuper rice”. No significant improvement of this parameter was observed in the placebo group. Furthermore, “Zuper rice” at a dose of 2 g/day also failed to produce a significant change in facial wrinkles.

### 2.3. Safety and Adverse Effects Evaluation

Table 3 and Table 4 show that there were no significant differences in hematological parameters among the groups either before the intervention or throughout the 12-week study period. When compared to the baseline, the placebo-treated group exhibited a significant increase in MCV (*p* < 0.05) and a slight decrease in MCHC (*p* < 0.01) after 12 weeks of treatment. However, these values were still in normal ranges. Subjects who consumed “Zuper rice” at doses of 2 g/day or 4 g/day showed no significant changes in any hematological parameters (the detailed changes in hematological parameters are provided as Supplementary Material File S2).

The blood chemistry values of the subjects in all treatment groups showed slightly high cholesterol before commencing the intervention, and all interventions used in this study failed to produce a significant change in this parameter, as shown in Table 4. The placebo-treated group exhibited significantly decreased T3 and T4, but increased TSH (*p*-value < 0.05 all, compared to the baseline). After 6 months of consumption, subjects who consumed “Zuper rice” at a dose of 2 g/day exhibited significantly decreased bicarbonate but increased AST from the baseline (*p*-value < 0.01 all), and, when the treatment was prolonged to 12 months, the significant elevation in AST disappeared, whereas the reduction in bicarbonate still presented (*p*-value < 0.05, compared to the baseline). Furthermore, a reduction in T4 was also observed (*p*-value < 0.05, compared to the baseline). Subjects who consumed “Zuper rice” at a dose of 4 g/day showed a decrease in creatinine after 6 months of consumption, and this change, together with an increase in HDL-Cholesterol, was observed after 12 months of consumption (*p*-value < 0.05, compared to the baseline). All changes observed in this study remained in the normal range, except AI, which has no normal range. All interventions failed to show the significant change from baseline AI. The details can be seen in Supplementary Material File S3.

It was found that, after 6 weeks of consumption, one participant who consumed the low dose and one participant who consumed the high dose of “Zuper rice” showed mild bloating, but this sign disappeared at 12 weeks of consumption. However, one participant in the placebo consumption group also showed signs of mild bloating. Both at 6 and 12 weeks of consumption, one case of low-dose consumption of “Zuper rice” showed a high and moderate degree of rash development, respectively. After 6 weeks of consumption, one case each of both high and low doses of “Zup” consumption reported mild diarrhea. In addition, one case in the low dose of “Zup” consumption group and one case in the placebo group reported a mild degree of stomach-ache.

### 2.4. Cardiovascular Risk

The AIP, a cardiovascular risk indicator, of subjects in the various treatment groups are revealed in Table 5. All subjects in this study were at high cardiovascular risk because their AIP > 0.24. The current data revealed that, in the placebo group, all subjects still showed AIP > 0.24 or were still in the high-risk group after 12 weeks of treatment. When compared to the baseline data, no significant change was observed. “Zuper rice” at a dose of 2 g/day showed that the AIP of this group still remained in the high-risk category for cardiovascular diseases after 6 weeks of treatment, and the AIP showed an intermediate risk for cardiovascular risk (AIP 0.24; intermediate range was 0.11–0.24) [41] when the intervention was prolonged to 12 months. However, the level of change was still not significant. Interestingly, the AIP of subjects who consumed “Zuper rice” at a dose of 4 g per day decreased to intermediate risk and nearly showed low risk (AIP = 0.22), and this change was significant (*p*-value < 0.05; compared to the baseline). Owing to this information, “Zuper rice” seemed to show a trend of improving cardiovascular risk, particularly the high dose of “Zuper rice”. (The details can be seen in Supplementary Material File S8).

### 2.5. Changes in Oxidative Stress and Inflammatory Markers

Table 6 shows the changes in 8-OGdG, which served as an oxidative stress marker, and TNF-α, NF-kB and IL-6, which served as inflammatory markers, as shown in Table 7. No significant differences in any of the parameters just mentioned among the groups were observed before the intervention. When compared to the baseline data, subjects who consumed a high dose of “Zuper rice” showed significantly decreased 8-OHdG at the 12-week consumption period (*p*-value < 0.05), while no significant change in this parameter was detected in the placebo- and low dose of “Zuper rice”-treated groups. No significant changes in any inflammatory markers were detected before the intervention. At 6 weeks of consumption, subjects who consumed “Zuper rice” at a dose of 2 g/day showed increased NF-kB, but this value significantly decreased when the intervention was prolonged to 12 weeks (*p*-value < 0.05 all; compared to the baseline). At 12 weeks of consumption, “Zuper rice” at doses of 2 and 4 g/day produced a significant increase in IL-6 (*p*-value < 0.01 and *p*-value < 0.001; compared to the baseline). No other significant changes were detected.

### 2.6. Changes in Telomere Length and Telomerase Activity

The effect of “Zuper rice” on the alterations in telomere length and telomerase are demonstrated in Table 8. Before the intervention, no significant differences in telomere length and telomerase among the various treatment groups were detected. At the end of a 12-week consumption period, subjects who consumed “Zuper rice” at a dose of 2 g/day showed significantly increased telomere length and telomerase activity (*p*-value < 0.01 and 0.05, respectively, compared to the baseline data).

## 3. Discussion

The present data revealed that a 12-week consumption of “Zuper rice” at 2 g/day increased the % accuracy of response in choice reaction time and a reduced response time in spatial memory, whereas “Zuper rice” consumption at a dose of 4 g/day increased the % accuracy of response in digit vigilance tests. A high dose of “Zuper rice” also decreased facial wrinkles and improved AIP. Increased telomere length and telomerase activity, together with a reduction in NF-kB and an increase in IL-6, were observed in subjects who consumed a low dose of “Zuper rice”, and a reduction in 8-OHdG and an increase in IL-6 were observed in subjects who consumed a high dose of “Zuper rice”. In addition, no changes in hematological or clinical chemistry values indicative of toxicity were observed. Some adverse effects were observed in both the placebo and “Zuper rice” consumption groups. However, the project physician has already explored this and found that the adverse effects that developed during the study period were not associated with the treatment.

A recent study has demonstrated the relationship between telomere length and cognitive function [42,43]. It has been revealed that better memory is associated with longer telomere length. Although the precise underlying mechanism remains unclear, it has been demonstrated that people with shorter telomeres are more vulnerable to neurodegeneration [44]. Therefore, the improved cognition induced by a low dose of “Zuper rice” may be attributed to decreased telomere attrition, leading to reduced neurodegeneration. In this study, telomerase also increased in the leukocytes of this treated group. Overall, the increase in telomerase in this group may be responsible for the reduction in telomere attrition [45], giving rise to the increase in telomere length and better cognition. In addition to telomere length, cognition is also associated with cognitive decline.

A large body of evidence also demonstrates that the nuclear factor-κB (NF-kB) signal pathway plays a crucial role in cognitive function. The NF-kB signalling pathway increases the synthesis and release of inflammatory cytokines, leading to an inflammatory response that, in turn, decreases synaptic plasticity and neuronal survival, resulting in cognitive decline [46,47,48]. Our data showed that a low dose of “Zuper rice” created a reduction in NF-kB. Therefore, the improvement in cognition may occur through reduced inflammation induced by NF-kB [46,47,48,49]. Therefore, the improvement in cognition observed in subjects who consumed a low dose of “Zuper rice” may be associated with the improvement in telomere length, telomerase and NF-kB.

The current data revealed that all subjects in this study showed elevated IL-6. However, significant elevation was observed in subjects who consumed “Zuper rice” at doses of 2 and 4 g per day. It has been revealed that IL-6 can be stimulated by NF-KB [50], which, in turn, is stimulated by TNF-α [51]. However, our study showed elevated IL-6 levels but decreased NF-kB levels. In addition, no close relation exists between the parameters mentioned above. This discrepancy may be due to the complex relationship between these parameters. It has been shown that, beyond NF-kB, many factors can increase IL-6, such as (1) age and health conditions, such as older age, obesity, diabetes, tissue injury, stress, chronic infection and inflammation [52]; (2) lifestyle and environmental factors, such as diet, exercise and pollution [53,54,55,56]; and (3) medical treatment, such as cancer treatment [57]. Our results show that there were no significant differences in age, weight, obesity, diabetes, tissue injury, stress, chronic infection and inflammation or exercise from the baseline. In addition, no cancer drug was used in this case. Thus, it is less likely to be the cause of the IL-6 elevation. Therefore, the possible causes for the IL-6 elevation in this study may be related to air pollution, such as PM2.5 [58], and diet, especially diets with a high dietary inflammatory index (DII) [59]. Owing to these possible causes, the reduction in NF-kB induced by “Zuper rice” cannot overcome the effects of air pollution and the consumption of a diet with high DII, and so the subjects who consumed “Zuper rice” show an elevation in IL-6. This aspect still requires further exploration to determine the precise underlying mechanism. Given the negative correlation between the DII score and serum IL-6 [60], this investigation should also control for DII during the intervention. Moreover, to identify and rule out the role of PM2.5 exposure, which can enhance IL-6, an investigation into the specific biomarkers of PM2.5, such as lysophosphatidylcholines (LysoPCs) [61], should also be conducted. Therefore, a larger sample size should also be used and analyzed using a retrograde approach to study their relationships, followed by an analysis of the differences between groups.

The brain is vulnerable to oxidative stress [62], which in turn induces neurodegeneration, particularly in the hippocampus and frontal cortex, which play crucial roles in cognitive function [63]. Neurodegeneration in the aforementioned areas can lead to cognitive decline. Based on this information, we suggest that the cognitive-enhancing effect of “Zuper rice” at a dose of 4 g/day may be partly associated with a reduction in oxidative stress, as indicated by a reduction in 8-OHdG.

The present results demonstrate that different areas show different susceptibilities to the possible active ingredient of “Zuper rice” or their metabolites, giving rise to an improvement of brain activity only in some areas, resulting in the improvement in some aspects of cognition, such as attention, sustained attention and the ability to understand language and recognize words. In addition, a low dose appears to show a high sensitivity to pathways involved in attention, while a high dose appears to engage the more complex network of word recognition. The difference in susceptibility may partly be due to differences in the bioavailability of the active substance in various areas. Furthermore, different doses of a drug can stimulate different receptors or pathways in the brain, leading to differences in brain responses across various tests in this study.

Oxidative stress plays an important role not only in neurodegeneration but also in wrinkle formation [64]. It can degrade collagen, disturb signal pathways and inhibit collagen synthesis. Oxidative stress can stimulate metalloproteinase (MMP), which in turn induces fragmentation of collagen fibrils. In addition, it inhibits TGF-β signalling, resulting in the inhibition of collagenous extracellular matrix (ECM) production [65]. Thus, the improvement in skin ageing in subjects who consumed high doses of “Zuper rice” in this study may be associated with the reduction in oxidative stress, which in turn improves the degradation of collagen and the activation of MMP, and which in turn gives rise to the reduction in ECM, including collagen and elastin production. Unfortunately, the detection of MMP was not performed in this study and appears to be a limitation.

Beyond the positive modulation effect of “Zuper rice” on cognitive function and facial wrinkles, a high dose of “Zuper rice” also improves AIP, an indicator of cardiovascular risk. It has been revealed that AIP is associated with heightened cardiovascular risk. Oxidative stress can induce the destruction of cells, including endothelial cells, leading to damage and dysfunction, contributing to atherosclerosis and other cardiovascular disorders [66,67]. Thus, the reduction in AIP which was observed in subjects who consumed high doses of “Zuper rice” for 12 weeks may be partly associated with oxidative stress.

The “Zuper rice” used in this study is rich in anthocyanins and amino acids, which are reputed for their anti-age-related effects, such as reduced cognitive decline, facial wrinkles and cardiovascular disease risk, and so the health benefits observed in this study may be associated with the availability of these substances. In addition, this study fails to show a dose-dependent effect, because each observed parameter is influenced by multiple factors and many pathways, making it less likely to observe a direct linear relationship between the concentration of “Zuper rice,” which consists of many ingredients, and the observed parameters. Moreover, when the concentration of “Zuper rice” was raised, the concentrations of all ingredients, not only the concentrations of possible active ingredients, but also the concentrations of inactive ingredients, were raised. This phenomenon can induce a masking effect. Owing to the involvement of multiple factors and pathways, along with the masking effect of the inactive ingredient, no dose-dependent effect was observed. Therefore, the high dose of “Zuper rice” fails to show significant changes in some parameters, such as telomere attrition and telomerase activity, whereas the low dose does. The placebo failed to show a positive modulation effect, because the placebo is only white rice that is subjected to heat similar to “ Zuper rice” and packed in a similar capsule as “Zuper rice”, but it has no anthocyanins and amino acids that play a role in cognition promotion, and so it fails to increase telomerase activity, telomere length and cognition. Accumulating lines of evidence also demonstrate that a cluster of amino acids can recruit telomerase [68,69], suppress NF-KB [70] and decrease oxidative stress [71]. Furthermore, anthocyanins can also inhibit NF-kB [72] and decrease oxidative stress [73]. Moreover, anthocyanins and amino acids can improve cognition [15,16,17,29,74], facial wrinkles [20,21,75,76] and AI [77,78]. Owing to the biological activities and the health benefits of anthocyanins and the cluster of amino acids mentioned above, we suggest that the anti-age-related disorder benefits mentioned earlier should be partly associated with the anthocyanins and amino acids contained in “Zuper rice”. This may be due to the direct effect of each substance or to interactions among various ingredients; further investigation is required. However, identifying the precise active substance requires further investigation of the metabolome.

Overall, our data reveal that both low and high doses of “Zuper rice” can enhance cognition. The mechanisms of the cognitive-enhancing effect of low-dose “Zuper rice” appear to involve the improvement in telomerase and telomere length, together with the reduction in inflammatory markers such as NF-KB, which in turn decreases inflammation, resulting in enhanced cognition manifested by an improvement in the % accuracy response of choice reaction time and a reduction in time response in a picture recognition test. In contrast, the cognitive-enhancing effect of the high dose of “Zuper rice” appears to involve improvements in oxidative stress and inflammation, as shown by the reduction in 8-OHdG, which, in turn, may involve brain plasticity and result in improved cognition, as manifested by an improvement in % accuracy in digit vigilance. The improvement in oxidative stress and inflammation induced by a high dose of “Zuper rice” may also reduce collagen degradation and MMP activity, leading to an improvement in facial wrinkles. Furthermore, they may reduce the opportunity for foam cell formation and the disturbance of endothelial function, resulting in an improvement in AIP. Our results from a 12-week study of “Zuper rice” consumption fail to show any toxicity signs. However, subjects who consumed a high dose of “Zuper rice” showed a significant increase in moderate and light exercise, as shown in Supplementary Material File S4. These types of exercise factors can also increase cognition [71]. However, at 12 weeks of consumption, subjects who consumed a high dose of “Zuper rice” and showed significant improvements in the digit vigilance task failed to show a significant increase in cognition, whereas both types of exercise showed significant increases. Owing to the discrepancy in cognitive and exercise changes, the physical activity changes in this group do not introduce any confounding error into our data. Furthermore, the data regarding physical activity in subjects who consumed a low dose of “Zuper rice” also fail to show a corresponding change in working memory at 6 and 12 weeks of consumption. Therefore, it is less likely to produce a confounding error on the effect of an intervention on memory. In addition, no significant change in food consumption was observed throughout the study (Supplementary Material File S5), suggesting that it is less likely to confound results. However, our study has the limitation that the food intake has not been analyzed in depth to determine the change in DII and DAQ-S. Although the improvement in cognition in this study appears to point to an association with the “Zuper rice” intervention, further research to control DII and DAQ-S should be performed to confirm the beneficial effects of anthocyanin- and anti-ageing amino acids-enriched pigmented rice.

## 4. Materials and Methods

### 4.1. Preparation of a Capsule Containing Heated “Zuper Rice”

“Zuper rice” was the rice mixture of three varieties of rice or *Oryza sativa,* including Khao Kum, Khao Hom Nil and Khao Rice Berry at an appropriate ratio (under petty patent registration), and this ratio provided high contents of anthocyanin and protein. All rice varieties used in this study were harvested from the Chaiyaphum and Roi-et provinces, Thailand, during November–December 2021. Owing to the cognitive-enhancing effect of anthocyanin-enriched substances at the doses of 2 and 4 g/day obtained from our previous study [79], both doses were applied to assess the effect of “Zuper rice” in this study.

**Figure 3:**
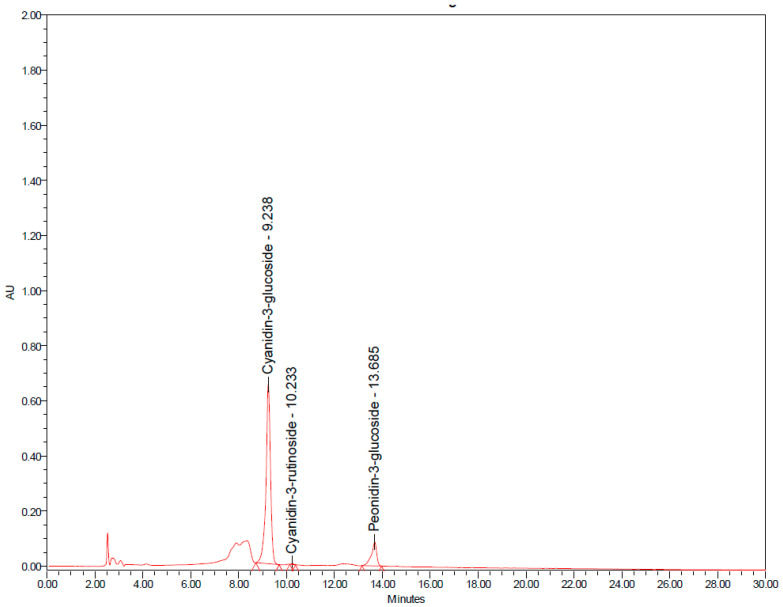
The anthocyanin profile of “Zuper rice”.

“Zuper rice” was heated at 90 °C for 30 min because it is the same temperature as that to which rice is subjected during the cooking process, and the anthocyanin content at this condition is the same as that of cooked “Zuper rice” (detailed information is provided in Supplementary Material File S6). The heated rice was ground to a powder with a herbal grinder, filtered through a 100-mesh sieve, and packed in dark green capsule number 00 (size 1000 mg) by TS Herb Products Co., Ltd., Sung Men, Phrae province, Thailand. Finally, the heated “Zuper rice” contained 1.731 ± 0.017 mg/g dry weight of anthocyanins. Thus, the doses of anthocyanins in the “Zuper rice” capsules used in this study were approximately 3500–7000 mg per day. The main anthocyanins presented in heated “Zuper rice” were assessed using a gradient elution system under the following conditions: mobile phase A consisted of 10% formic acid in deionized water (10:90, *v*/*v*), and mobile phase B consisted of acetonitrile with a flow rate of 1.0 mL/min, where the injection volume was 20 µL. Detection was performed at a wavelength of 530 nm. The concentrations of cyanidin-3 glucoside, peonidin-3-glucoside and cyanidin-3-rutinoside were 0.616 ± 0.074, 0.193 ± 0.014 and 0.002 ± 0.000 mg/g of heated “Zuper rice”, respectively, as shown in Figure 3. The placebo was prepared from Hom Mali white rice subjected to a heating process similar to “Zuper rice”. The placebo and the capsule containing “Zuper rice” were identical.

### 4.2. Ethical Statement

This study was a single-centre, 3-arm, 12-week, randomized, double-blind and placebo-controlled trial, and it was approved by the Khon Kaen Ethical Committee for Human Research. It was approved on 30 November 2022 (HE651411). This study was also registered in the Thai Clinical Trial Registry (TCTR20221017004) on 12 October 2022. All procedures were performed in accordance with the International Conference of Harmonization (ICH) for Good Clinical Practice (GCP) and in compliance with the Declaration of Helsinki and its subsequent amendments. The study site was established at the Faculty of Medicine, Khon Kaen University (approval date: 21 November 2022). Written informed consent from all subjects was obtained prior to their participation in this study.

### 4.3. Study Participants

The recruitment was performed via advertisement. All potential participants were first screened by staff using a structured interview, followed by a physical examination by the project physician.

Sample size

The sample size in this study was calculated based on a randomized clinical trial sample size calculation formula. The values of type one error (α) and type two error (β) were set at 0.05 and 0.20 to obtain 80% power. Based on our previous work [79], the standard deviation was considered to be 2.64, and the effect of size was 2.14. According to the calculation, the sample size per group was around 24. We expected a 20% dropout rate, or five people per group. Therefore, 30 participants per group were used. This value corresponds with an acceptable sample size for a clinical trial when the significance level is set at 5% (one-sided) to obtain 80% power [80,81,82].

Participant eligibility

Eligibility included males and females aged 45–65 years, with body mass index (BMI) 18–25 kg/m^2^, and participants with a stress level evaluated by the Depression Anxiety Stress Scale-21 (DASS-21) of less than 7 were screened by a physician and well-trained researchers. In addition, they did not regularly consume a probiotic diet and had to be able to communicate in Thai. They were excluded if they met any of the following criteria: (1) people with current treatment for cardiovascular disease, lung, metabolic or endocrine disorders, cancer, or liver or kidney disease; (2) a history of head trauma, brain disorders or mental disorders; (3) currently pregnant or lactating or continually taking a contraceptive pill; (4) alcoholic beverage consumption greater than five glasses/day; (5) smoking more than 10 pieces/day of cigar; (6) current medication or dietary supplement use that may exert an influence on brain function; and (7) current participation in other projects.

Randomization and blinding

Participants were randomized into placebo, low-dose (2 g/day) or high-dose (4 g/day) treatment groups at a ratio of 1:1:1 by using simple randomization generated by computer and performed by a member of staff who was not involved in the study. In this study, neither the participants nor the researchers knew who was receiving a placebo or control, and who was receiving the active intervention. Allocation and randomization were hidden until the final analyses were completed. The code was kept in an opaque envelope at the principal indicator and could be opened after data analysis or before it, if a medical emergency occurred.

### 4.4. Experimental Protocol

Male and female middle-aged volunteers aged 45–65 were recruited from Amphoe Muaeng, Khon Kaen province, mainly via an announcement. They were screened for eligibility using an interview and physical examination. After screening, participants who met the eligibility criteria were randomly assigned to placebo, low-dose (2 g/day), or high-dose (4 g/day) treatment groups by a member of staff who was not involved in the study. The dosage range of “Zuper rice” used in this study was selected based on previously reported anthocyanin doses that revealed a cognitive-enhancing effect via modulation of monoamine oxidase [15]. All subjects had to consume white rice for 2 weeks before participating in this study. The participants had to consume the assigned substance for 12 weeks. All subjects in this study were assessed for primary outcome and telomere length, and secondary outcomes including cognition, skin ageing, consumption safety, cardiovascular risk, telomerase and oxidative stress and inflammatory markers before the intervention and every 6 weeks throughout the study period. Subjects could withdraw at any time, and if serious side effects occurred then the study was stopped. The detailed of this randomized clinical trial was provided in Supplementary Material File S7.

### 4.5. Cognitive Assessment

The crucial cognitive function measured—the ability to hold information temporarily in a heightened state of availability for use in ongoing information processing [83], which plays a crucial role in operating information for tasks like reasoning, problem-solving and learning—was determined by using a battery of recognition tests, including a word recognition test, picture recognition test, simple reaction test, digit vigilance test, choice reaction time test, spatial test and numeric working memory test [15]. In brief, the subjects were instructed to perform each task, and the reaction time, together with the percentage of accurate responses, was recorded. According to this battery test, the response time of the simple reaction test, choice reaction time and digit vigilance served as indices of attentional power, whereas the percentage of response accuracy in digit vigilance and choice reaction time served as indices of attentional continuity. Response times in word and picture recognition tests, together with spatial and numeric working memory tests, were used as indices of memory speed, while the percentage of accurate responses in these tests was used as an index of memory quality.

### 4.6. Facial Wrinkle Assessment

In this study, we assessed ageing facial skin using a skin analyser (ASL, Anti-ageing Scale for the Face 100 model, Aram Huvis Co., Ltd., Gyeonggi-do, 13,605, Seongnam city, Republic of Korea). Both sides of the temple, cheek and area around the eye were assessed for hydration, elasticity and wrinkles. The processes were performed according to the company’s instructions. The assessment involved taking standardized facial photographs and analyzing them to assess various signs of ageing, including wrinkles. Grading performance was assessed using the grading system outlined by Michaels et al. [84]. The facial wrinkle score was obtained by combining scores from multiple measurements, modified from the Fitzpatrick wrinkle classification system [85]. The grading score ranged from 0 to 3 (0 = absent, 0.5 = shallow wrinkle, 1 = wrinkle with slight indentation, 1.5 = wrinkle and clear indentation less than 1 mm in depth, 2 = wrinkle 1 to 2 mm in depth, 2.5 = wrinkle more than 2 mm and up to 3 mm in depth and 3 = wrinkle more than 3 mm in depth) by two blinded researchers by comparison with reference photographs. Data were obtained from the summation of facial wrinkles and presented as mean ± SD. Higher scores indicated a higher level of wrinkling.

### 4.7. Biochemical Assessments

We assessed the effects of “Zuper rice” on inflammation by using the serum levels of tumour necrosis factor-α (TNF-α), Interleukin 6(IL-6) and nuclear factor-kappa B (NF-kB). In addition, 8-hydroxy-2′-deoxyguanosine (8-OHdG) was used as an oxidative stress marker.

Serum 8-OHdG level was assessed by using an ELISA kit (ab28524, from Abcam Limited, Cambridge, UK) according to the manufacturer’s instructions. All reagents used in this study were freshly prepared according to the instructions. The 96-well microplate was washed two times with 1X Wash Solution before adding 50 μL of each of the following substances: standard, sample and control. Then, they were allowed to react with 50 μL of Biotinylated Detection Antibody working solution for 45 min under a 37 °C incubation period. Then, the solution was discarded, and the sample was washed three times with 1X Wash Solution. After washing, all residual wash liquid was removed, and the sample was allowed to react with 0.1 mL of Streptavidin-Biotinylated Horseradish Peroxidase Complex (SABC) working solution at 37 °C for 30 min. The solution was then discarded, and the sample was washed five times with 1X Wash Solution. Following this step, it was allowed to react with 90 L of 3,3′,5,5′-Tetramethylbenzidine (TMB) substrate at 37 °C for 15–30 min until a shade of blue was observed in the first 3–4 wells. Then, the reaction was stopped with 50 L of stop solution, and the absorbance at 450 nm was measured within 20 min [79].

ELISA kits were used to detect serum levels of TNF-α (ab108908 from Abcam Limited, Cambridge, UK) and IL-6 (ab178013 from Abcam Limited, Cambridge, UK) according to the method described in our previous study [79].

Measurement of serum NF-kB was performed by using a commercial kit (CSB-E12107h, Cusabio, Cuba Innovation Center, Houston, TX, USA). All assay procedures were carried out according to the company’s instructions. In brief, an aliquot of standard or sample was added to each well, covered with the provided adhesive strip, and incubated at 37° for 2 h. At the end of incubation, the fluid was discarded, 100 μL of Biotin-antibody (1×) was added and the sample was covered with a new adhesive strip and incubated for 1 h at 37 °C. Then, each well was aspirated, washed by filling it with 200 L of wash buffer and left to stand for 2 min. Following this step, any remaining wash Buffer was removed by aspirating or decanting, the plate was inverted and it was blotted against clean paper towels. Following this step, an aliquot of HRP-avidin (1×) at a volume of 100 μL was added to each well, the plate was covered with a new adhesive strip and it was incubated at 37 °C for 1 h. The aspiration and washing process was repeated five times. After washing, 90 μL of TMB Substrate was added to each well and incubated in dark conditions for 15–30 min at 37 °C. At the end of incubation, a stop solution of 50 L was added to each well, and the mixture was mixed thoroughly by tapping gently. Then, absorbance at 450 nm was determined.

### 4.8. Assessments of Telomere Length and Telomerase Enzyme Activity

Buffy coats from all subjects were collected, and DNA was extracted using the GF-1 Nucleic acid extraction kit (Vivantis Technologies Sdn Bhd, Selangor Darul Ehsan, Malaysia). In brief, 200 µL of Binding Buffer (BB) was mixed with 200 µL of plasma sample in a microcentrifuge tube, vortexed thoroughly, and then 20 µL of Proteinase K was added, vortexed again and incubated at 65 °C for 10 min. At the end of incubation, 20 µL of RNase A (DNase-Free, 20 mg/mL) was added, mixed and incubated for 10 min at 37 °C to remove RNA. Then, absolute ethanol at a volume of 200 L was added, thoroughly mixed to produce a homogeneous solution and loaded into the column, which was assembled in a provided clean collection tube. Following this step, the solution was centrifuged at 5000× *g* for 1 min. Then, the flow-through was discarded, and the column was washed with 500 L of wash buffer 1, then centrifuged at 5000× *g* for 1 min. The flow-through was discarded again, and the sample was rewashed with 500 L of wash buffer 2 and centrifuged at maximum speed for 3 min. After centrifugation, DNA was eluted from the column and placed into a clean microcentrifuge tube. Then, 100 L of the preheated Elution Buffer was directly added to the column membrane, allowed to stand for 2 min and centrifuged at 5000× *g* for 1 min to elute DNA. The obtained DNA was quantified using a Thermo Scientific^TM^ NanoDrop spectrophotometer (Fisher Scientific, NH, USA). The DNA extraction was stored at −80 °C until used.

To amplify DNA, a PCR was implemented. In brief, the PCR mixture with a total volume of 25 μL was mixed with 12.5 μL of SYBR^®^ Green PCR Master Mix (2×) (Thermofisher Scientific, MA, USA); 8.5 μL of PCR-grade water; 1 μL (2 μM/L) of each telomere primer, such as telomere forward (*teloF*) (Integrate DNA, Technologies, Coralville, IA, USA) and telomere reward (*teloR*) (Integrate DNA, Technologies, Coralville, IA, USA); single-copy genes such as 36B4 Forward *(36B4F)* (Integrate DNA, Technologies, Coralville, IA, USA) and 36B4 Reward (*36B4R*) (Integrate DNA, Technologies, Coralville, IA, USA); and 2 μL of DNA (5 ng/μL sample). In this study, 36B4 served as a reference gene encoding acidic ribosomal phosphoprotein. The nucleotide sequences of all primers mentioned above are shown in Table 9. Then, a real-time system (Roter-Gene, QIAGEN, Germantown, MD, USA) was implemented for the amplification. The thermal cycling of *36B4* included one cycle (95 °C for 15 s), followed by 40 cycles (95 °C for 15 s, 57 °C for 1 min), whereas telomere cycling included one cycle (95 °C for 10 min), followed by 50 cycles (95 °C for 15 s, 58 °C for 1 min [86,87].

The calculation of telomere length (TL) was performed by using a provided equation [88]:

TL (kb) = 3.274 + 2.413 × (T/S)

The T/S ratio or telomere gene/single-copy gene ratio was calculated from the cycle threshold (Ct) of telomere and single copy gene (36B4) according to the following formula previously described [37,38]:

ΔCt telomere = [Ct (telomere of DNA sample) − Ct (telomere of DNA control)]

∆Ct single copy gene = [Ct (single copy gene of DNA sample) − Ct (single copy gene of DNA control)]

∆∆Ct sample = [∆Ct telomere − ∆Ct single copy gene]

Relative telomere (T/S ratio) = 2 − ∆∆Ct sample

Telomerase activity was measured using a commercial kit (MBS3802216; MyBiosource, San Diego, CA, USA) according to the manufacturer’s instructions. All reagents were freshly prepared prior to use. Briefly, 50 µL of each telomerase standard was added to the designated wells for the standard curve. For sample wells, 10 µL of sample was added, followed by 40 µL of sample diluent. Next, 100 µL of HRP-conjugate reagent was added to every well; the plate was sealed and incubated at 37 °C for 60 min. After incubation, wells were aspirated and washed five times with 400 µL of wash solution. Residual wash buffer was removed by inverting the plate and blotting it on clean paper towels. Then, 50 µL each of the chromogen solutions A and B were added to each well, and the plate was gently tapped to mix, then incubated in the dark at 37 °C for 15 min. Finally, 50 µL of stop solution was added to each well, and absorbance was measured at 450 nm within 15 min.

### 4.9. Safety Evaluation and Adverse Effect Assessment

To ensure the safety of consumption for a pigmented rice innovation, changes in hematological and clinical chemistry parameters were determined by the Srinagarind Hospital Excellence Laboratory, Faculty of Medicine, Khon Kaen University. Adverse effects, including the appearance and severity (mild, moderate and severe) of nausea and vomiting, stomach-ache, bloating and rash were also recorded.

### 4.10. Cardiovascular Risk Assessment

In this study, we evaluated cardiovascular risk using the atherogenic index in plasma (AIP), a validated marker [89]. The AIP was calculated as described below and expressed in mmol/L:

AIP = log (TG/HDL-C)

### 4.11. Statistical Analysis

In this study, a statistical analysis of effectiveness was performed on the per-protocol (PP) set. Only the data obtained from subjects who completed all visits were analyzed. Data are expressed as mean ± SD. All data were tested for normality using the Shapiro–Wilk test. Data that showed a normal distribution were tested for statistical differences using a repeated measures ANOVA (one-tailed) and a Tukey post hoc test. For data that failed to show normality, the Kruskal–Wallis test was used for statistical analysis. In this study, cognitive data were compared with the placebo group, whereas the other parameters were compared with the baseline data. A statistical difference was determined when *p*-value < 0.05.

## 5. Conclusions

This study is the first to reveal the beneficial health effects of the pigmented rice formulation “Zuper rice”, and its role in telomere and telomerase modulation, as well in reducing oxidative stress and inflammation. Consuming “Zuper rice” at doses of 2 and 4 g/day improves cognition. The possible underlying mechanism may depend on the dosage. A low dose (2 g/d) may exert this effect by increasing telomerase, which in turn decreases telomere attrition, together with a reduction in NF-kB, thereby improving cognition, whereas a high dose might involve a reduction in oxidative stress. “Zuper rice” at a dose of 4 g/day also reduces cardiovascular risk and improves facial wrinkles. The mechanisms behind this finding may partly involve reduced oxidative stress. These observed health benefits may be attributed to the anthocyanins (1.638 and 3.276 mg per day) and amino acids present in “Zuper rice”. No toxicity or serious side effects are observed after the ingestion of “Zuper rice” for 12 weeks. Therefore, our results demonstrate that “Zuper rice” is a potential pigmented rice innovation that can be used to promote healthy ageing. However, further studies with a larger sample size and investigations into the effects on collagen and ECM alterations should be conducted to determine the mechanism behind its anti-facial wrinkle effects.

## Figures and Tables

**Table 1:** General characteristics of subjects (*n* = 30/gr). Data are expressed as mean ± SD.

Parameters	Baseline	6-Week	12-Week
**Placebo (*n* = 30)**			
Age (years)	52.80 ± 0.99	52.80 ± 0.99 (*p* = 1.000)	53.00 ± 1.01 (*p* = 1.000)
Gender (male/female)	3/27	3/27	3/27
Blood temperature (°C)	36.60 ± 0.02	36.66 ± 0.02 (*p* = 0.547)	36.61 ± 0.02 (*p* = 1.000)
Heart rate (beats/min)	71.53 ± 1.48	71.93 ± 1.84 (*p* = 1.000)	70.62 ± 1.13 (*p* = 0.739)
Respiratory rate (breaths/min)	17.03 ± 0.09	17.03 ± 0.06 (*p* = 1.000)	17.34 ± 0.10 (*p* = 0.143)
Systolic BP (mmHg)	119.23 ± 2.10	117.10 ± 1.96 (*p* = 0.645)	117.62 ± 1.91 (*p* = 0.266)
Diastolic BP (mmHg)	70.83 ± 2.08	71.93 ± 1.92 (*p* = 1.000)	72.83 ± 2.04 (*p* = 0.352)
Body weight (kg)	57.22 ± 1.18	56.86 ± 1.16 (*p* = 0.344)	57.26 ± 1.10 (*p* = 0.318)
Body height (cm)	156.93 ± 1.02	156.93 ± 1.02 (*p* = 1.000)	157.17 ± 1.03 (*p* = 1.000)
BMI (kg/m^2^)	23.21 ± 0.39	23.08 ± 0.04 (*p* = 0.379)	23.18 ± 0.40 (*p* = 0.339)
**Zuper rice 2 g/day (*n* = 30)**			
Age (years)	52.03 ± 0.88	52.03 ± 0.88 (*p* = 1.000)	52.03 ± 0.88 (*p* = 1.000)
Gender (male/female)	3/27	3/27	3/27
Blood temperature (°C)	36.58 ± 0.02	36.60 ± 0.02 (*p* = 1.000)	36.61 ± 0.03 (*p* = 1.000)
Heart rate (beats/min)	71.00 ± 1.05	72.67 ± 1.27 (*p* = 0.669)	73.37 ± 1.09 (*p* = 0.161)
Respiratory rate (breaths/min)	17.00 ± 0.07	17.10 ± 0.09 (*p* = 0.791)	16.97 ± 0.14 (*p* = 1.000)
Systolic BP (mmHg)	115.00 ± 1.85	111.10 ± 1.92 (*p* = 0.063)	110.90 ± 1.93 * (*p* = 0.031)
Diastolic BP (mmHg)	67.70 ± 1.79	69.33 ± 1.57 (*p* = 0.371)	66.80 ± 1.37 (*p* = 1.000)
Body weight (kg)	56.90 ± 1.17	56.90 ± 1.14 (*p* = 1.000)	57.11 ± 1.15 (*p* = 0.505)
Body height (cm)	157.13 ± 0.90	157.13 ± 0.90 (*p* = 1.000)	157.13 ± 0.90 (*p* = 1.000)
BMI (kg/m^2^)	23.05 ± 0.45	23.06 ± 0.44 (*p* = 1.000)	23.15 ± 0.45 (*p* = 0.449)
**Zuper rice 4 g/day (*n* = 30)**			
Age (years)	52.27 ± 0.99	52.27 ± 0.99 (*p* = 1.000)	52.27 ± 0.99 (*p* = 1.000)
Gender (male/female)	3/27	3/27	3/27
Blood temperature (°C)	36.63 ± 0.03	36.62 ± 0.02 (*p* = 1.000)	36.62 ± 0.02 (*p* = 1.000)
Heart rate (beats/min)	69.63 ± 1.22	69.83 ± 1.35(*p* = 1.000)	69.77 ± 1.36 (*p* = 1.000)
Respiratory rate (breaths/min)	16.97 ± 0.08	17.23 ± 0.09(*p* = 0.054)	17.23 ± 0.10 (*p* = 0.090)
Systolic BP (mmHg)	117.23 ± 2.25	114.00 ± 2.16(*p* = 0.271)	113.50 ± 2.15 (*p* = 0.105)
Diastolic BP (mmHg)	70.53 ± 1.66	69.17 ± 1.67 (*p* = 0.860)	69.77 ± 1.17 (*p* = 1.000)
Body weight (kg)	55.69 ± 0.97	55.31 ± 1.02 (*p* = 0.247)	55.24 ± 1.03 (*p* = 0.075)
Body height (cm)	154.73 ± 0.91	154.73 ± 0.91 (*p* = 1.000)	154.73 ± 0.91 (*p* = 1.000)
BMI (kg/m^2^)	23.26 ± 0.35	23.10 ± 0.37 (*p* = 0.252)	23.06 ± 0.37 (*p* = 0.062)

Data are presented as mean ± standard deviation (SD). * *p*-value < 0.05, compared to the placebo control group.

**Table 2:** Response time and percentage of accuracy response of the computerized battery test in various intervention groups, including placebo, “Zuper rice” 2 and 4 g per day-treated groups (*n* = 30/gr). Data are expressed as mean ± SD.

Parameters		Placebo (*n* = 30)	Zuper Rice (2 g/day) (*n* = 30)	Zuper Rice (4 g/day) (*n* = 30)
**Baseline**				
Word Recognition	Time	1345.90 ± 81.37	1360.34 ± 77.05 (*p* = 0.826)	1361.39 ± 82.38 (*p* = 0.826)
	%Accuracy	85.73 ± 2.04	86.74 ± 1.85 (*p* = 0.740)	86.79 ± 1.64 (*p* = 0.953)
Picture Recognition	Time	1425.40 ± 79.63	1389.78 ± 64.60 (*p* = 0.940)	1412.89 ± 71.94 (*p* = 0.983)
	%Accuracy	86.80 ± 1.43	84.46 ± 1.61 (*p* = 0.272)	83.40 ± 1.77 (*p* = 0.165)
Simple Reaction	Time	664.49 ± 26.76	659.13 ± 23.20 (*p* = 1.000)	681.48 ± 25.99 (*p* = 0.734)
Digit Vigilance	Time	663.19 ± 10.09	669.03 ± 8.75 (*p* = 0.735)	660.58 ± 11.32 (*p* = 0.778)
	%Accuracy	92.61 ± 0.91	94.23 ± 0.82 (*p* = 0.179)	93.33 ± 1.04 (*p* = 0.459)
Choice Reaction Time	Time	835.89 ± 20.80	836.95 ± 17.92 (*p* = 0.967)	826.47 ± 15.14 (*p* = 0.719)
	%Accuracy	98.24 ± 0.46	98.14 ± 0.41 (*p* = 0.702)	97.68 ± 0.86 (*p* = 1.000)
Spatial Memory	Time	1451.34 ± 57.83	1351.73 ± 45.77 (*p* = 0.186)	1469.67 ± 58.10 (*p* = 0.815)
	%Accuracy	90.99 ± 2.18	91.76 ± 1.87 (*p* = 0.747)	84.77 ± 1.48 (*p* = 0.213)
Numeric Working Memory	Time	1169.06 ± 43.17	1114.13 ± 37.86 (*p* = 0.376)	1171.36 ± 33.95 (*p* = 0.798)
	%Accuracy	92.53 ± 2.09	96.66 ± 1.13 (*p* = 0.072)	95.60 ± 1.25 (*p* = 0.309)
**6 weeks**				
Word Recognition	Time	1145.57 ± 34.68	1153.29 ± 39.92 (*p* = 0.889)	1193.42 ± 39.29 (*p* = 0.376)
	%Accuracy	86.01 ± 2.06	90.52 ± 1.75 (*p* = 0.086)	89.09 ± 1.64 (*p* = 0.340)
Picture Recognition	Time	1394.51 ± 82.71	1266.91 ± 40.87 (*p* = 0.339)	1382.52 ± 77.40 (*p* = 0.706)
	%Accuracy	87.75 ± 1.79	88.68 ± 1.86 (*p* = 0.730)	86.59 ± 1.98 (*p* = 0.739)
Simple Reaction	Time	688.08 ± 38.58	660.53 ± 30.02 (*p* = 0.536)	648.74 ± 16.19 (*p* = 0.378)
Digit Vigilance	Time	674.27 ± 11.58	675.71 ± 9.89 (*p* = 0.966)	677.60 ± 7.15 (*p* = 0.772)
	%Accuracy	90.25 ± 1.22	93.45 ± 1.34 (*p* = 0.083)	93.53 ± 0.85 * (*p* = 0.036)
Choice Reaction Time	Time	907.73 ± 48.24	825.00 ± 21.65 (*p* = 0.071)	840.50 ± 14.75 (*p* = 0.127)
	%Accuracy	96.80 ± 0.63	98.52 ± 0.47 * (*p* = 0.035)	98.00 ± 0.39 (*p* = 0.164)
Spatial Memory	Time	1428.15 ± 67.40	1411.57 ± 90.03 (*p* = 0.555)	1437.83 ± 51.67 (*p* = 0.687)
	%Accuracy	95.14 ± 1.31	96.63 ± 1.07 (*p* = 0.476)	95.57 ± 1.24 (*p* = 0.843)
Numeric Working Memory	Time	1205.42 ± 87.48	1122.24 ± 43.40 (*p* = 0.329)	1147.51 ± 32.35 (*p* = 0.480)
	%Accuracy	94.50 ± 1.95	97.36 ± 1.09 (*p* = 0.429)	95.15 ± 1.70 (*p* = 1.000)
**12 weeks**				
Word Recognition	Time	1236.37± 81.34	1170.18 ± 70.53 (*p* = 0.765)	1281.69± 66.01 (*p* = 0.451)
	%Accuracy	89.55 ± 1.83	88.71 ± 2.34 (*p* = 0.871)	90.63 ± 1.46 (*p* = 0.636)
Picture Recognition	Time	1397.87 ± 81.59	1189.39 ± 61.84 * (*p* = 0.018)	1287.25 ± 48.33 (*p* = 0.255)
	%Accuracy	88.33 ± 1.59	86.15 ± 2.66 (*p* = 0.603)	87.61 ± 1.64 (*p* = 0.908)
Simple Reaction	Time	753.56 ± 74.11	663.18 ± 45.56 (*p* = 0.259)	676.90 ± 25.02 (*p* = 0.395)
Digit Vigilance	Time	677.55 ± 14.10	660.62 ± 14.70 (*p* = 0.279)	670.76 ± 9.57 (*p* = 0.422)
	%Accuracy	91.36 ± 1.46	94.59 ± 1.33 (*p* = 0.129)	92.67 ± 1.09 (*p* = 0.487)
Choice Reaction Time	Time	893.01 ± 49.60	831.73 ± 28.73 (*p* = 0.420)	869.68 ± 21.26 (*p* = 0.692)
	%Accuracy	98.40 ± 0.40	98.15 ± 0.47 (*p* = 0.713)	98.28 ± 0.39 (*p* = 0.959)
Spatial Memory	Time	1409.67 ± 95.82	1273.87 ± 75.84 (*p* = 0.475)	1497.62 ± 76.97 (*p* = 0.328)
	%Accuracy	96.10 ± 1.29	96.36 ± 1.45 (*p* = 0.785)	94.84 ± 1.50 (*p* = 0.825)
Numeric Working Memory	Time	1194.36 ± 73.14	1093.55 ± 47.35 (*p* = 0.596)	1127.09 ± 30.52 (*p* = 0.885)
	%Accuracy	94.00 ± 1.92	95.38 ± 1.75 (*p* = 0.612)	94.76 ± 1.18 (*p* = 0.987)

* *p*-value < 0.05, compared to the placebo control group.

**Table 3:** The hematological parameters of subjects in the three groups: placebo and “Zuper rice” at doses of 2 and 4 g per day, at the baseline and after 6 and 12 weeks of consumption (*n* = 30/gr). Data are expressed as mean ± SD.

Parameters	Reference	Baseline	6-Week	12-Week
**Placebo (*n* = 30)**				
RBC	4.7–6.2 10^6^/μL	4.57 ± 0.07	4.68 ± 0.06 (*p* = 0.154)	4.57 ± 0.06 (*p* = 1.000)
MCV	80.0–97.8 fL	86.27 ± 1.25	85.83 ± 1.18 (*p* = 0.962)	87.49 ± 1.27 * (*p* = 0.035)
MCH	25.2–32.0 pg	27.60 ± 0.46	27.33 ± 0.42 (*p* = 0.096)	27.63 ± 0.44 (*p* = 1.000)
MCHC	31.3–33.4 g/dL	31.96 ± 0.12	31.84 ± 0.15 (*p* = 1.000)	31.57 ± 0.15 ** (*p* = 0.004)
RDW	11.9–14.8%	13.66 ± 0.22	13.50 ± 0.19 (*p* = 0.253)	13.63 ± 0.22 (*p* = 1.000)
**Zuper rice 2 g/day (*n* = 30)**				
RBC	4.7–6.2 10^6^/μL	4.70 ± 0.10	4.75 ± 0.12 (*p* = 1.000)	4.81 ± 0.11 (*p* = 0.107)
MCV	80.0–97.8 fL	81.49 ± 2.02	81.33 ± 2.08 (*p* = 1.000)	81.12 ± 2.06 (*p* = 0.504)
MCH	25.2–32.0 pg	25.43 ± 0.69	25.49 ± 0.71 (*p* = 1.000)	25.30 ± 0.71 (*p* = 1.000)
MCHC	31.3–33.4 g/dL	31.16 ± 0.21	31.29 ± 0.18 (*p* = 1.000)	31.15 ± 0.24 (*p* = 1.000)
RDW	11.9–14.8%	14.68 ± 0.43	14.70 ± 0.43 (*p* = 1.000)	14.51 ± 0.42 (*p* = 0.310)
**Zuper rice 4 g/day (*n* = 30)**				
RBC	8.7–12.5 fL	4.58 ± 0.09	4.67 ± 0.09 (*p* = 0.346)	4.63 ± 0.10 (*p* = 1.000)
MCV	4.7–6.2 10^6^/μL	84.11 ± 1.95	83.34 ± 1.93 (*p* = 0.665)	84.06 ± 1.93 (*p* = 0.755)
MCH	80.0–97.8 fL	26.66 ± 0.68	26.32 ± 0.67 (*p* = 0.504)	26.43 ± 0.67 (*p* = 1.000)
MCHC	25.2–32.0 pg	31.65 ± 0.22	31.57 ± 0.22 (*p* = 1.000)	31.41 ± 0.22 (*p* = 0.317)
RDW	31.3–33.4 g/dL	14.38 ± 0.46	14.24 ± 0.42 (*p* = 0.665)	14.19 ± 0.46 (*p* = 0.394)

*, ** *p*-value < 0.05 and 0.01, respectively, compared to the baseline of each group.

**Table 4:** Blood chemistry parameters of subjects in the three groups: placebo and “Zuper rice” at doses of 2 and 4 g per day, at the baseline and after 6 and 12 weeks of consumption (*n* = 30/gr). Data are expressed as mean ± SD.

Parameters	Reference	Baseline	6-Week	12-Week
**Placebo (*n* = 30)**				
BUN	5.8–19.1 mg/dL	12.14 ± 0.57	11.85 ± 0.56 (*p* = 1.000)	11.65 ± 0.45 (*p* = 0.896)
Creatinine	0.5–1.5 mg/dL	0.83 ± 0.02	0.81 ± 0.02 (*p* = 0.994)	0.80 ± 0.02 (*p* = 0.215)
Sodium	130–147 mEq/L	139.07 ± 0.30	139.40 ± 0.37 (*p* = 0.264)	139.07 ± 0.34 (*p* = 1.000)
Potassium	3.4–4.7 mEq/L	4.50 ± 0.06	4.38 ± 0.08 (*p* = 0.360)	4.51 ± 0.10 (*p* = 1.000)
Bicarbonate	20.6–28.3 mEq/L	22.40 ± 0.54	21.94 ± 0.30 (*p* = 1.000)	22.96 ± 0.38 (*p* = 0.653)
Chloride	96–107 mEq/L	101.27 ± 0.37	101.30 ± 0.50 (*p* = 1.000)	101.79 ± 0.40 (*p* = 0.465)
Albumin	3.8–5.4 g/dL	4.37 ± 0.04	4.47 ± 0.04 ** (*p* = 0.003)	4.39 ± 0.04 (*p* = 1.000)
Total Bilirubin	0.3–1.5 mg/dL	0.49 ± 0.07	0.61 ± 0.08 (*p* = 0.053)	0.52 ± 0.07 (*p* = 1.000)
ALT	4–36 U/L	16.47 ± 1.21	15.77 ± 1.15 (*p* = 1.000)	17.90 ± 1.92 (*p* = 1.000)
AST	12–32 U/L	21.47 ± 1.04	22.43 ± 1.10 (*p* = 0.647)	23.48 ± 1.75 (*p* = 0.406)
ALP	42–121 U/L	78.60 ± 7.28	74.23 ± 3.98 (*p* = 1.000)	78.24 ± 4.52 (*p* = 1.000)
Thyroxine (T4)	4.5–11.7 μg/dL	7.30 ± 0.31	7.00 ± 0.24 (*p* = 0.838)	6.61 ± 0.22 * (*p* = 0.011)
Triiodothyronine (T3)	80–200 ng/dL	108.25 ± 3.22	110.33 ± 4.17 (*p* = 1.000)	99.44 ± 3.00 * (*p* = 0.012)
Cholesterol	Less than 200 mg/dL	206.47 ± 5.23	216.20 ± 7.44 (*p* = 0.093)	212.34 ± 6.66 (*p* = 0.173)
Triglyceride	10–200 mg/dL	120.83 ± 11.42	114.60 ± 7.08 (*p* = 1.000)	124.59 ± 11.19 (*p* = 1.000)
HDL-Chol	>35 mg/dL	59.37 ± 2.63	61.87 ± 3.04 (*p* = 0.206)	61.62 ± 3.05 (*p* = 0.241)
AI	-	2.48 ± 0.16	2.49 ± 0.14 (*p* = 1.000)	2.44 ± 0.15 (*p* = 1.000)
LDL-Chol (DIRECT)	10–150 mg/dL	134.50 ± 5.04	143.77 ± 6.75 * (*p* = 0.038)	137.14 ± 5.97 (*p* = 0.833)
**Zuper rice 2 g/day (*n* = 30)**				
BUN	5.8–19.1 mg/dL	10.47 ± 0.56	10.53 ± 0.53 (*p* = 1.000)	10.73 ± 0.51 (*p* = 1.000)
Creatinine	0.5–1.5 mg/dL	0.78 ± 0.01	0.79 ± 0.02 (*p* = 1.000)	0.77 ± 0.01 (*p* = 0.641)
Sodium	130–147 mEq/L	138.87 ± 0.44	139.40 ± 0.37 (*p* = 0.765)	138.80 ± 0.42 (*p* = 1.000)
Potassium	3.4–4.7 mEq/L	4.55 ± 0.09	4.51 ± 0.07 (*p* = 1.000)	4.47 ± 0.06 (*p* = 1.000)
Bicarbonate	20.6–28.3 mEq/L	22.62 ± 0.35	21.03 ± 0.37 ** (*p* = 0.001)	21.43 ± 0.35 * (*p* = 0.016)
Chloride	96–107 mEq/L	101.23 ± 0.37	101.80 ± 0.35 (*p* = 0.596)	101.67 ± 0.32 (*p* = 1.000)
Albumin	3.8–5.4 g/dL	4.33 ± 0.05	4.35 ± 0.05 (*p* = 1.000)	4.31 ± 0.05 (*p* = 1.000)
Total Bilirubin	0.3–1.5 mg/dL	0.47 ± 0.04	0.49 ± 0.04 (*p* = 0.795)	0.51 ± 0.04 (*p* = 0.482)
ALT	4–36 U/L	16.60 ± 1.35	19.23 ± 1.59 (*p* = 0.053)	21.57 ± 2.69 (*p* = 0.102)
AST	12–32 U/L	22.07 ± 1.35	24.93 ± 1.36 ** (*p* = 0.007)	24.73 ± 1.78 (*p* = 0.139)
ALP	42–121 U/L	72.10 ± 4.23	74.87 ± 4.50 (*p* = 0.193)	79.505 ± 5.22 ** (*p* = 0.004)
Thyroxine (T4)	4.5–11.7 μg/dL	7.30 ± 0.32	6.78 ± 0.30 (*p* = 0.207)	6.57 ± 0.22 ** (*p* = 0.001)
Triiodothyronine (T3)	80–200 ng/dL	107.52 ± 3.98	108.35 ± 4.12 (*p* = 1.000)	102.08 ± 3.43 (*p* = 0.158)
Cholesterol	Less than 200 mg/dL	206.93 ± 5.94	209.43 ± 6.80 (*p* = 1.000)	210.37 ± 6.17 (*p* = 0.765)
Triglyceride	10–200 mg/dL	113.77 ± 7.70	115.87 ± 8.94 (*p* = 1.000)	112.33 ± 7.45 (*p* = 1.000)
HDL-Chol	>35 mg/dL	60.70 ± 2.71	63.37 ± 3.38 (*p* = 0.202)	63.63 ± 3.25 (*p* = 0.084)
AI	-	2.45 ± 0.13	2.30 ± 0.16 *p* = 1.000)	2.31 ± 0.16 (*p* = 1.000)
LDL-Chol (DIRECT)	10–150 mg/dL	135.40 ± 5.52	135.67 ± 5.95 (*p* = 1.000)	138.00 ± 5.73 (*p* = 1.000)
**Zuper rice 4 g/day (*n* = 30)**				
BUN	5.8–19.1 mg/dL	11.59 ± 0.67	11.09 ± 0.62 (*p* = 0.958)	11.09 ± 0.59 (*p* = 0.855)
Creatinine	0.5–1.5 mg/dL	0.78 ± 0.01	0.76 ± 0.01 * (*p* = 0.012)	0.75 ± 0.01 ** (*p* = 0.002)
Sodium	130–147 mEq/L	138.93 ± 0.30	138.93 ± 0.26 (*p* = 1.000)	138.73 ± 0.34 (*p* = 1.000)
Potassium	3.4–4.7 mEq/L	4.63 ± 0.09	4.44 ± 0.05 (*p* = 0.186)	4.38 ± 0.06 (*p* = 0.071)
Bicarbonate	20.6–28.3 mEq/L	21.39 ± 0.54	21.43 ± 0.39 (*p* = 1.000)	22.72 ± 0.41 (*p* = 0.193)
Chloride	96–107 mEq/L	102.10 ± 0.29	101.50 ± 0.39 (*p* = 0.178)	101.27 ± 0.34 (*p* = 0.195)
Albumin	3.8–5.4 g/dL	4.29 ± 0.04	4.33 ± 0.04 (*p* = 0.950)	4.32 ± 0.04 (*p* = 0.989)
Total Bilirubin	0.3–1.5 mg/dL	0.40 ± 0.04	0.47 ± 0.03 (*p* = 0.151)	0.46 ± 0.03 (*p* = 0.457)
ALT	4–36 U/L	17.13 ± 1.07	16.13 ± 1.16 (*p* = 0.999)	17.17 ± 1.76 (*p* = 1.000)
AST	12–32 U/L	23.27 ± 1.00	22.90 ± 0.86 (*p* = 1.000)	23.13 ± 1.06 (*p* = 1.000)
ALP	42–121 U/L	72.10 ± 4.76	72.47 ± 4.23 (*p* = 1.000)	74.73 ± 4.33 (*p* = 1.000)
Thyroxine (T4)	4.5–11.7 μg/dL	6.74 ± 0.21	6.84 ± 0.23 (*p* = 1.000)	6.46 ± 0.19 (*p* = 0.226)
Triiodothyronine (T3)	80–200 ng/dL	106.05 ± 3.08	112.83 ± 4.45 (*p* = 0.177)	109.90 ± 4.63 (*p* = 0.796)
Cholesterol	Less than 200 mg/dL	200.87 ± 6.16	203.73 ± 7.22 (*p* = 1.000)	206.43 ± 6.22 (*p* = 0.662)
Triglyceride	10–200 mg/dL	115.97 ± 11.46	117.07 ± 14.32 (*p* = 1.000)	103.00 ± 10.52 (*p* = 0.131)
HDL-Chol	>35 mg/dL	57.50 ± 2.69	59.30 ± 2.78 (*p* = 0.489)	61.57 ± 2.90 * (*p* = 0.039)
AI	-	2.49 ± 0.19	2.43 ± 0.20 (*p* = 0.785)	2.35 ± 0.16 (*p* = 0.117)
LDL-Chol (DIRECT)	10–150 mg/dL	129.77 ± 5.54	131.43 ± 5.96 (*p* = 1.000)	134.07 ± 5.35 (*p* = 0.869)

*, ** *p*-value < 0.05 and 0.01, respectively, compared to the baseline of each group.

**Table 5:** Atherogenic index in plasma (AIP) of subjects in the placebo and “Zuper rice” groups, at doses of 2 and 4 g per day (*n* = 90). Data are presented as mean ± SD.

Atherogenic Index In Plasma (AIP)	References	Placebo	“Zuper Rice” 2 g/day	“Zuper Rice” 4 g/day
Baseline	<0.11	0.31 ± 0.07	0.27 ± 0.05	0.30 ± 0.06
6-week	<0.11	0.27 ± 0.05 (*p* = 1.000)	0.26 ± 0.09 (*p* = 0.950)	0.26 ± 0.06 (*p* = 0.334)
12-week	<0.11	0.30 ± 0.06 (*p* = 0.666)	0.24 ± 0.07 (*p* = 1.000)	0.22 ± 0.06 * (*p* = 0.030)

* *p*-value < 0.05, compared to baseline data.

**Table 6:** Oxidative stress markers of subjects in the placebo and “Zuper rice” groups, at doses of 2 and 4 g per day (*n* = 90). Data are presented as mean ± SD.

8-OHdG (ng/mL)	Baseline	6-Week	12-Week
Placebo (*n* = 30)	199.71 ± 13.51	199.20 ± 12.33 (*p* = 1.000)	140.43 ± 20.76 (*p* = 0.059)
Zuper rice 2 g/day (*n* = 30)	204.91 ± 17.02	199.07 ± 18.87 (*p* = 0.330)	172.20 ± 20.63 (*p* = 0.433)
Zuper rice 4 g/day (*n* = 30)	243.62 ± 17.47	244.85 ± 18.56 (*p* = 0.109)	157.62 ± 17.18 * (*p* = 0.031)

* *p*-value < 0.05, compared to the baseline of each group.

**Table 7:** Inflammatory markers of subjects in the placebo and “Zuper rice” groups, at doses of 2 and 4 g per day (*n* = 90). Data are presented as mean ± SD.

Parameters	Baseline	6-Week	12-Week
**NF-kB (ng/mL)**			
Placebo (*n* = 30)	3.37 ± 0.29	4.29± 0.50 (*p* = 0.108)	2.34 ± 0.18 (*p* = 0.071)
Zuper rice 2 g/day (*n* = 30)	3.67 ± 0.26	4.92 ± 0.53 * (*p* = 0.031)	2.29 ± 0.16 ** (*p* = 0.004)
Zuper rice 4 g/day (*n* = 30)	3.14 ± 0.33	3.42 ± 0.63 (*p* = 1.000)	2.31 ± 0.19 (*p* = 0.295)
**TNF-α** **(ng/mL)**			
Placebo (*n* = 30)	3.25 ± 0.72	4.56 ± 1.69 (*p* = 0.464)	4.73 ± 0.40 (*p* = 0.565)
Zuper rice 2 g/day (*n* = 30)	5.70 ± 3.12	5.19 ± 1.67 (*p* = 1.000)	4.91 ± 0.42 (*p* = 0.306)
Zuper rice 4 g/day (*n* = 30)	1.56 ± 0.65	2.35 ± 0.68 (*p* = 0.890)	4.64 ± 0.25 (*p* = 0.451)
**IL-6** **(pg/mL)**			
Placebo (*n* = 30)	11.43 ± 3.99	10.57 ± 2.31 (*p* = 1.000)	38.24 ± 14.76 (*p* = 0.389)
Zuper rice 2 g/day (*n* = 30)	12.73 ± 2.44	15.53 ± 2.35 (*p* = 0.639)	21.70 ± 1.00 ** (*p* = 0.006)
Zuper rice 4 g/day (*n* = 30)	7.53 ± 1.76	8.40 ± 1.82 (*p* = 1.000)	24.30 ± 1.59 *** (*p* < 0.001)

*, ** *p*-value < 0.05 and 0.01, respectively; ***: *p* < 0.001, compared to the baseline of each group.

**Table 8:** Telomere length and telomerase activity of subjects in the placebo group and the “Zuper rice” intervention groups, both doses of 2 and 4 g per day (*n* = 30/arm). Data are presented as mean ± SD.

Parameters	Placebo (*n* = 30)	Zuper Rice 2 g/day (*n* = 30)	Zuper Rice 4 g/day (*n* = 30)
**Telomere Length**			
Baseline	4.96 ± 0.05	4.98 ± 0.07	4.98 ± 0.06
6-week	5.02 ± 0.08 (*p* = 1.000)	4.95 ± 0.05 (*p* = 1.000)	4.97 ± 0.08 (*p* = 1.000)
12-week	5.23 ± 0.31 (*p* = 1.000)	5.46 ± 0.24 ** (*p* = 0.006)	4.55 ± 0.35 (*p* = 0.629)
**Telomerase (ng/mL)**			
Baseline	13.29 ± 0.89	14.27 ± 0.67	12.32 ± 0.84
6-week	11.83 ± 1.08 (*p* = 0.165)	12.80 ± 1.05 (*p* = 0.326)	10.51 ± 1.46 (*p* = 0.681)
12-week	16.09 ± 0.90 (*p* = 0.102)	17.57 ± 0.87 * (*p* = 0.035)	15.70 ± 0.87 (*p* = 0.078)

*, ** *p*-value < 0.05 and 0.01, respectively, compared to the baseline of each group.

**Table 9:** The types of primer and sequence of nucleotides of the telomere primer and 36B4 primer (the information regarding the nucleic acid sequence is provided in Supplementary Material S8).

PCR Primers	Oligomer Sequence (5′–3′)	Amplicon Size
*teloF*	CGGTTTGTTTGGGTTTGGGTTTGGGTTTGGG TTTGGGTT	>76 bp
*teloR*	GGCTTGCCTTACCCTTACCCTTACCC TTACCCTTACCCT	
*36B4F*	CAGCAAGTGGGAAGGTGTAATCC	75 bp
*36B4R*	CCCATTCTATCATCAACGGGTACAA	

## Data Availability

The data presented in this study are available on request from the corresponding author.

## Supplementary Materials

The following supporting information can be downloaded at: https://www.mdpi.com/article/10.3390/ijms262210911/s1 [90].
