# Supplementary material for: An Anthocyanin- and Anti-Ageing Amino Acids-Enriched Pigmented Rice Innovation Promotes Healthy Ageing Through the Modulation of Telomere, Oxidative Stress and Inflammation Reduction: A Randomized Clinical Trial
Source: Int J Mol Sci. 2025 Nov 11;26(22):10911. doi: 10.3390/ijms262210911 (PMC12652741; doi:10.3390/ijms262210911)
Supplement: Supplementary file 1 [file ijms-26-10911-s001.zip › Supplementary material file S2 Hematological changes of subjects.pdf]

Supplementary material file S2 . The hematological parameters of subjects in various groups including placebo, "Zuper rice "at doses of 2 and 4 g per day at baseline and after 6 and 12 weeks of consumption. (N=30/gr) Data were expressed as mean ± SD.

**Parameters**		**Baseline**	**6-week**	**12-week**
**Placebo (*n*=30)**	Reference			
Hb	13.0-16.7 g/dL	12.56±0.21	12.76±0.19 (p=0.398)	12.59±0.21 (p=1.000)
HCT	40.5-50.8 %	39.26±0.58	40.07±0.57 (p=0.266)	39.88±0.60 (p=0.369)
WBC	4.6-10.6 10^3/µl	6.62±0.28	6.62±0.26 (p=1.000)	6.47±0.30 (p=1.000)
Platelets	173-383 10^3/µl	289.03±12.45	275.23±9.20 (p=0.460)	276.52±10.35 (p=0.288)
MPV	8.7-12.5 fL	10.23±0.16	10.28±0.15 (p=1.000)	10.09±0.15 (p=0.680)
RBC	4.7-6.2 10^6/µl	4.57±0.07	4.68±0.06 (p=0.154)	4.57±0.06 (p=1.000)
MCV	80.0-97.8 fL	86.27±1.25	85.83±1.18 (p=0.962)	87.49±1.27 *(p=0.035)
MCH	25.2-32.0 pg	27.60±0.46	27.33±0.42 (p=0.096)	27.63±0.44 (p=1.000)
MCHC	31.3-33.4 g/dL	31.96±0.12	31.84±0.15 (p=1.000)	31.57±0.15 **(p=0.004)
RDW	11.9-14.8 %	13.66±0.22	13.50±0.19 (p=0.253)	13.63±0.22 (p=1.000)
NE%	43.7-70.9 %	54.17±1.44	52.15±1.69 (p=0.754)	52.62±1.71 (p=1.000)
LY%	20.1-44.5 %	33.77±1.23	36.14±1.64 (p=0.463)	34.77±1.52 (p=1.000)
MO%	3.4-9.8 %	6.04±0.35	5.73±0.29 (p=0.814)	6.55±0.34 (p=0.594)
EO%	0.7-9.2 %	5.28±0.95	5.16±0.82 (p=1.000)	5.33±1.04 (p=1.000)
BA%	0.0-2.6 %	0.74±0.06	0.82±0.07 (p=0.570)	0.73±0.06 (p=1.000)
**Zuper rice 2 g/day (*n*=30)**				
Hb	13.0-16.7 g/dL	11.82±0.25	11.95±0.27 (p=1.000)	12.02±0.26 (p=0.493)
HCT	40.5-50.8 %	37.87±0.73	38.17±0.83 (p=1.000)	38.57±0.77 (p=0.391)
WBC	4.6-10.6 10^3/µl	6.52±0.27	6.65±0.35 (p=1.000)	6.36±0.24 (p=1.000)
Platelets	173-383 10^3/µl	294.03±9.96	285.93±11.89 (P=0.425)	300.47±11.97 (p=1.000)
MPV	8.7-12.5 fL	10.30±0.14	10.18±0.16 (p=0.548)	10.37±0.16 (p=0.941)
RBC	4.7-6.2 10^6/µl	4.70±0.10	4.75±0.12 (p=1.000)	4.81±0.11 (p=0.107)
MCV	80.0-97.8 fL	81.49±2.02	81.33±2.08 (p=1.000)	81.12±2.06 (p=0.504)
MCH	25.2-32.0 pg	25.43±0.69	25.49±0.71 (p=1.000)	25.30±0.71 (p=1.000)
MCHC	31.3-33.4 g/dL	31.16±0.21	31.29±0.18 (p=1.000)	31.15±0.24 (p=1.000)
RDW	11.9-14.8 %	14.68±0.43	14.70±0.43 (p=1.000)	14.51±0.42 (p=0.310)
NE%	43.7-70.9 %	54.34±1.65	54.06±1.99 (p=1.000)	53.22±1.57 (p=1.000)
LY%	20.1-44.5 %	35.29±1.42	35.29±1.72 (p=1.000)	35.98±1.39 (p=1.000)
MO%	3.4-9.8 %	5.45±0.25	5.52±0.18 (p=1.000)	5.80±0.26 (p=0.192)
EO%	0.7-9.2 %	4.11±0.58	4.26±0.62 (p=1.000)	4.11±0.52 (p=1.000)
BA%	0.0-2.6 %	0.82±0.07	0.87±0.08 (p=0.604)	0.85±0.08 (p=1.000)
**Zuper rice 4 g/day (*n*=30)**				
Hb	13.0-16.7 g/dL	12.07±0.19	12.17±0.23 (p=0.478)	12.10±0.18 (p=1.000)
HCT	40.5-50.8 %	38.10±0.47	38.54±0.63 (p=0.292)	38.52±0.47 (p=0.429)
WBC	4.6-10.6 10^3/µl	6.31±0.30	6.46±0.30 (p=1.000)	6.43±0.30 (p=1.000)
Platelets	173-383 10^3/µl	274.60±11.39	274.83±11.33 (p=1.000)	284.86±9.96 (p=0.562)
MPV	8.7-12.5 fL	10.45±0.18	10.27±0.18 (p=0.067)	10.16±0.15 (p=0.170)
RBC	4.7-6.2 10^6/µl	4.58±0.09	4.67±0.09 (p=0.346)	4.63±0.10 (p=1.000)
MCV	80.0-97.8 fL	84.11±1.95	83.34±1.93 (p=0.665)	84.06±1.93 (p=0.755)
MCH	25.2-32.0 pg	26.66±0.68	26.32±0.67 (p=0.504)	26.43±0.67 (p=1.000)
MCHC	31.3-33.4 g/dL	31.65±0.22	31.57±0.22 (p=1.000)	31.41±0.22 (p=0.317)
RDW	11.9-14.8 %	14.38±0.46	14.24±0.42 (p=0.665)	14.19±0.46 (p=0.394)
NE%	43.7-70.9 %	51.13±1.43	52.59±1.33 (p=0.432)	52.63±1.33 (p=0.787)
LY%	20.1-44.5 %	37.29±1.28	35.81±1.21 (p=0.368)	36.07±1.16 (p=0.929)

MO%	3.4-9.8 %	5.49±0.30	5.58±0.24 (p=1.000)	5.50±0.22 (p=1.000)
EO%	0.7-9.2 %	5.28±0.58	5.21±0.55 (p=1.000)	4.98±0.52 (p=0.876)
BA%	0.0-2.6 %	0.82±0.07	0.82±0.07 (p=1.000)	0.80±0.06 (p=1.000)

*, ** p-value < 0.05 and 0.01 respectively, compared to the baseline of each group.
