# Supplementary material for: An Anthocyanin- and Anti-Ageing Amino Acids-Enriched Pigmented Rice Innovation Promotes Healthy Ageing Through the Modulation of Telomere, Oxidative Stress and Inflammation Reduction: A Randomized Clinical Trial
Source: Int J Mol Sci. 2025 Nov 11;26(22):10911. doi: 10.3390/ijms262210911 (PMC12652741; doi:10.3390/ijms262210911)
Supplement: Supplementary file 1 [file ijms-26-10911-s001.zip › Supplementary material file S3 Blood chemistry parameters of subjects in various groups including placebo.pdf]

Supplementary material file S3 Blood chemistry parameters of subjects in various groups including placebo, "Zuper rice " at doses of 2 and 4 g per day at baseline and after 6 and 12 weeks of consumption. (N=30/gr) Data were expressed as mean ± SD.

Parameters		Baseline	6-week	12-week
**Placebo (*n*=30)**	Reference			
BUN	5.8-19.1 mg/dL	12.14±0.57	11.85±0.56 (p=1.000)	11.65±0.45 (p=0.896)
Creatinine	0.5-1.5 mg/dL	0.83±0.02	0.81±0.02 (p=0.994)	0.80±0.02 (p=0.215)
Uric Acid	2.7-7.0 mg/dL	5.23±0.21	5.12±0.18 (p=0.623)	5.04±0.20 (p=0.069)
Sodium	130-147 mEq/L	139.07±0.30	139.40±0.37 (p=0.264)	139.07±0.34 (p=1.000)
Potassium	3.4-4.7 mEq/L	4.50±0.06	4.38±0.08 (p=0.360)	4.51±0.10 (p=1.000)
Bicarbonate	20.6-28.3 mEq/L	22.40±0.54	21.94±0.30 (p=1.000)	22.96±0.38 (p=0.653)
Chloride	96-107 mEq/L	101.27±0.37	101.30±0.50 (p=1.000)	101.79±0.40 (p=0.465)
Total Protein	6.5-8.8 g/dL	7.57±0.10	7.69±0.09 (p=0.187)	7.62±0.09 (p=0.538)
Albumin	3.8-5.4 g/dL	4.37±0.04	4.47±0.04 **(p=0.003)	4.39±0.04 (p=1.000)
Globulin	2.6-3.4 g/dL	3.20±0.07	3.22±0.08 (p=1.000)	3.25±0.08 (p=0.760)
Total Bilirubin	0.3-1.5 mg/dL	0.49±0.07	0.61±0.08 (p=0.053)	0.52±0.07 (p=1.000)
Direct Bilirubin	0.0-0.5 mg/dL	0.19±0.02	0.21±0.02 (p=0.330)	0.19±0.02 (p=1.000)
ALT	4-36 U/L	16.47±1.21	15.77±1.15 (p=1.000)	17.90±1.92 (p=1.000)
AST	12-32 U/L	21.47±1.04	22.43±1.10 (p=0.647)	23.48±1.75 (p=0.406)
ALP	42-121 U/L	78.60±7.28	74.23±3.98 (p=1.000)	78.24±4.52 (p=1.000)
Amylase	25-125 U/L	84.37±5.49	84.80±5.72 (p=1.000)	87.86±6.06 (p=0.913)
LDH	0-250 U/L	201.73±6.46	200.40±6.23 (p=1.000)	201.52±7.98 (p=1.000)
CK	28-140 U/L	113.66±9.46	109.41±10.92 (p=1.000)	129.97±18.05 (p=1.000)
Gamma GT	0-50 U/L	28.53±4.09	25.07±3.41 (p=0.769)	29.31±5.34 (p=1.000)
Homocysteine	0-15U/L	11.20±0.61	10.77±0.64 (p=1.000)	10.14±0.64 (p=0.053)
Thyroxine (T4)	4.5-11.7 µg/dL	7.30±0.31	7.00±0.24 (p=0.838)	6.61±0.22 *(p=0.011)
Triiodothyronine (T3)	80-180 ng/dL	108.25±3.22	110.33±4.17 (p=1.000)	99.44±3.00 *(p=0.012)
TSH	80-180 ng/dl	1.87±0.29	1.99±0.25 (p=0.575)	2.18±0.27 *(p=0.036)
LH	14.2–52.3 IU/L	27.09±3.67	27.99±3.30 (p=0.356)	27.52±3.63 (p=1.000)
FSH	25.8 to 134.8 mIU/mL	53.98±7.41	53.97±6.53 (p=1.000)	53.92±7.33 (p=1.000)
Estradiol	12.4-398 pg/ml	43.79±18.10	51.58±22.78 (p=1.000)	40.15±12.45 (p=1.000)
Testosterone	1.93-8.36 ng/ml	5.81±1.79	7.69±1.99 (p=0.197)	5.77±1.67 (p=1.000)
Cortisol in Blood	6.2-19.4 ug/dL	8.11±0.49	7.74±0.54 (p=0.886)	7.54±0.67 (p=1.000)
Cholesterol	Less than 200 mg/dL	206.47±5.23	216.20±7.44 (p=0.093)	212.34±6.66 (p=0.173)
Triglyeride	10 - 200 mg/dL	120.83±11.42	114.60±7.08 (p=1.000)	124.59±11.19 (p=1.000)
HDL-Chol	>35 mg/dL	59.37±2.63	61.87±3.04 (p=0.206)	61.62±3.05 (p=0.241)
AI	-	2.63±0.16	2.63±0.14 (p=1.000)	2.60±0.15 (p=1.000)
LDL-Chol (DIRECT)	10-150 mg/dL	134.50±5.04	143.77±6.75 *(p=0.038)	137.14±5.97 (p=0.833)
**Zuper rice 2 g/day (*n*=30)**				

BUN	5.8-19.1 mg/dL	10.47±0.56	10.53±0.53 (p=1.000)	10.73±0.51 (p=1.000)
Creatinine	0.5-1.5 mg/dL	0.78±0.01	0.79±0.02 (p=1.000)	0.77±0.01 (p=0.641)
Uric Acid	2.7-7.0 mg/dL	4.86±0.20	4.90±0.19 (p=1.000)	5.03±0.21 (p=0.322)
Sodium	130-147 mEq/L	138.87±0.44	139.40±0.37 (p=0.765)	138.80±0.42 (p=1.000)
Potassium	3.4-4.7 mEq/L	4.55±0.09	4.51±0.07 (p=1.000)	4.47±0.06 (p=1.000)
Bicarbonate	20.6-28.3 mEq/L	22.62±0.35	21.03±0.37 **(p=0.001)	21.43±0.35 *(p=0.016)
Chloride	96-107 mEq/L	101.23±0.37	101.80±0.35 (p=0.596)	101.67±0.32 (p=1.000)
Total Protein	6.5-8.8 g/dL	7.57±0.09	7.67±0.07 (p=0.482)	7.70±0.06 (p=0.383)
Albumin	3.8-5.4 g/dL	4.33±0.05	4.35±0.05 (p=1.000)	4.31±0.05 (p=1.000)
Globulin	2.6-3.4 g/dL	3.24±0.08	3.32±0.07 (p=0.375)	3.39±0.06 (p=0.081)
Total Bilirubin	0.3-1.5 mg/dL	0.47±0.04	0.49±0.04 (p=0.795)	0.51±0.04 (p=0.482)
Direct Bilirubin	0.0-0.5 mg/dL	0.19±0.01	0.19±0.01 (p=1.000)	0.19±0.01 (p=1.000)
ALT	4-36 U/L	16.60±1.35	19.23±1.59 (p=0.053)	21.57±2.69 (p=0.102)
AST	12-32 U/L	22.07±1.35	24.93±1.36 **(p=0.007)	24.73±1.78 (p=0.139)
ALP	42-121 U/L	72.10±4.23	74.87±4.50 (p=0.193)	79.505±5.22 **(p=0.004)
Amylase	25-125 U/L	93.80±5.59	94.17±5.87 (p=1.000)	94.17±5.54 (p=1.000)
LDH	0-250 U/L	201.13±8.00	203.67±7.62 (p=1.000)	198.43±7.16 (p=1.000)
CK	28-140 U/L	119.47±9.77	113.22±10.14 (p=1.000)	117.70±9.50 (p=1.000)
Gamma GT	0-50 U/L	31.77±7.73	27.97±4.27 (p=0.556)	28.43±4.13 (p=0.148)
Homocysteine	0-15U/L	10.07±0.45	10.17±0.44 (p=1.000)	9.73±0.39 (p=0.947)
Thyroxine (T4)	4.5-11.7 μg/dL	7.30±0.32	6.78±0.30 (p=0.207)	6.57±0.22 **(p=0.001)
Triiodothyronine (T3)	80-180 ng/dL	107.52±3.98	108.35±4.12 (p=1.000)	102.08±3.43 (p=0.158)
TSH	80-180 ng/dl	1.85±0.17	1.97±0.21 (p=1.000)	1.84±0.17 (p=1.000)
LH	14.2–52.3 IU/L	22.99±3.04	23.17±3.34 (p=1.000)	29.84±3.43 (p=0.215)
FSH	25.8 to 134.8 mIU/m**L**	41.86±6.28	42.37±6.40 (p=1.000)	44.51±5.94 (p=1.000)
Estradiol	12.4-398 pg/ml	50.09±12.35	78.72±26.29 (p=1.000)	56.05±20.27 (p=0.931)
Testosterone	1.93-8.36 ng/ml	5.23±0.25	5.52±1.15 (p=1.000)	5.98±1.00 (p=1.000)
Cortisol in Blood	6.2-19.4 ug/dL	8.43±0.66	8.95±0.97 (p=1.000)	8.27±0.81 (p=1.000)
Cholesterol	Less than 200 mg/dL	206.93±5.94	209.43±6.80 (p=1.000)	210.37±6.17 (p=0.765)
Triglyceride	10 - 200 mg/dL	113.77±7.70	115.87±8.94 (p=1.000)	112.33±7.45 (p=1.000)
HDL-Chol	>35 mg/dL	60.70±2.71	63.37±3.38 (p=0.202)	63.63±3.25 (p=0.084)
AI	-	2.53±0.13	2.48±0.16 p=1.000)	2.50±0.16 (p=1.000)
LDL-Chol (DIRECT)	10-150 mg/dL	135.40±5.52	135.67±5.95 (p=1.000)	138.00±5.73 (p=1.000)
**Zuper rice 4 g/day (*n*=30)**				
BUN	5.8-19.1 mg/dL	11.59±0.67	11.09±0.62 (p=0.958)	11.09±0.59 (p=0.855)
Creatinine	0.5-1.5 mg/dL	0.78±0.01	0.76±0.01 *(p=0.012)	0.75±0.01 **(p=0.002)
Uric Acid	2.7-7.0 mg/dL	4.66±0.17	4.64±0.16 (p=1.000)	4.57±0.16 (p=1.000)
Sodium	130-147 mEq/L	138.93±0.30	138.93±0.26 (p=1.000)	138.73±0.34 (p=1.000)
Potassium	3.4-4.7 mEq/L	4.63±0.09	4.44±0.05 (p=0.186)	4.38±0.06 (p=0.071)

Bicarbonate	20.6-28.3 mEq/L	21.39±0.54	21.43±0.39 (p=1.000)	22.72±0.41 (p=0.193)
Chloride	96-107 mEq/L	102.10±0.29	101.50±0.39 (p=0.178)	101.27±0.34 (p=0.195)
Total Protein	6.5-8.8 g/dL	7.56±0.08	7.58±0.07 (p=1.000)	7.72±0.09 (p=0.071)
Albumin	3.8-5.4 g/dL	4.29±0.04	4.33±0.04 (p=0.950)	4.32±0.04 (p=0.989)
Globulin	2.6-3.4 g/dL	3.27±0.07	3.26±0.07 (p=1.000)	3.39±0.08 (p=0.060)
Total Bilirubin	0.3-1.5 mg/dL	0.40±0.04	0.47±0.03 (p=0.151)	0.46±0.03 (p=0.457)
Direct Bilirubin	0.0-0.5 mg/dL	0.16±0.02	0.19±0.01 (p=0.269)	0.18±0.01 (p=0.552)
ALT	4-36 U/L	17.13±1.07	16.13±1.16 (p=0.999)	17.17±1.76 (p=1.000)
AST	12-32 U/L	23.27±1.00	22.90±0.86 (p=1.000)	23.13±1.06 (p=1.000)
ALP	42-121 U/L	72.10±4.76	72.47±4.23 (p=1.000)	74.73±4.33 (p=1.000)
Amylase	25-125 U/L	97.34±6.09	101.07±6.42 (p=0.376)	98.07±5.93 (p=1.000)
LDH	0-250 U/L	205.07±9.09	189.73±5.48 (p=0.242)	191.47±7.98 (p=0.370)
CK	28-140 U/L	109.67±9.37	107.97±9.75 (p=1.000)	117.23±11.05 (p=1.000)
Gamma GT	0-50 U/L	30.23±8.33	25.33±5.09 (p=0.660)	26.40±4.42 (p=1.000)
Homocysteine	0-15U/L	9.93±0.31	9.90±0.38 (p=1.000)	9.43±0.40 (p=0.497)
Thyroxine (T4)	4.5-11.7 µg/dL	6.74±0.21	6.84±0.23 (p=1.000)	6.46±0.19 (p=0.226)
Triiodothyronine (T3)	80-180 ng/dL	106.05±3.08	112.83±4.45 (p=0.177)	109.90±4.63 (p=0.796)
TSH	80-180 ng/dl	1.94±0.16	1.73±0.16 (p=0.552)	1.91±0.17 (p=1.000)
LH	14.2–52.3 IU/L	30.19±3.71	32.83±4.01 (p=1.000)	34.88±5.14 (p=0.916)
FSH	25.8 to 134.8 mIU/mL	49.30±6.59	49.82±5.87 (p=1.000)	47.61±6.28 (p=1.000)
Estradiol	12.4-398 pg/ml	58.50±14.11	55.78±13.09 (p=1.000)	54.48±17.20 (p=1.000)
Testosterone	1.93-8.36 ng/ml		No male participants	
Cortisol in Blood	6.2-19.4 ug/dL	7.79±0.60	8.04±0.63 (p=1.000)	8.96±0.75 (p=0.202)
Cholesterol	Less than 200 mg/dL	200.87±6.16	203.73±7.22 (p=1.000)	206.43±6.22 (p=0.662)
Triglyceride	10 - 200 mg/dL	115.97±11.46	117.07±14.32 (p=1.000)	103.00±10.52 (p=0.131)
HDL-Chol	>35 mg/dL	57.50±2.69	59.30±2.78 (p=0.489)	61.57±2.90 *(p=0.039)
AI	-	2.69±0.19	2.61±0.20 (p=0.785)	2.51±0.16 (p=0.117)
LDL-Chol (DIRECT)	10-150 mg/dL	129.77±5.54	131.43±5.96 (p=1.000)	134.07±5.35 (p=0.869)

*, ** p-value < 0.05 and 0.01 respectively, compared to the baseline of each group.
