# Supplementary material for: An Anthocyanin- and Anti-Ageing Amino Acids-Enriched Pigmented Rice Innovation Promotes Healthy Ageing Through the Modulation of Telomere, Oxidative Stress and Inflammation Reduction: A Randomized Clinical Trial
Source: Int J Mol Sci. 2025 Nov 11;26(22):10911. doi: 10.3390/ijms262210911 (PMC12652741; doi:10.3390/ijms262210911)
Supplement: Supplementary file 1 [file ijms-26-10911-s001.zip › Supplementary material file S4 Physical activity zuper rice.pdf]

Supplementay material file S4

Table. Physical activity of volunteers who consumed placebo, or "Zuper rice" at the doses of 2, and 4 g per day at baseline. (N=30/arm). Data are expressed as mean±S.E.M.

Parameter		Baseline		
		Placebo (n=30)	Zuper rice 2 g/day (n=30)	Zuper rice 4 g/day (n=30)
high intensity activity	Run fast	0.00±0.00	0.03±0.03 (p=0.317)	0.27±0.14 (p=0.040)*
	Lifting heavy objects	1.27±0.43	0.93±0.40 (p=0.556)	0.50±0.27 (p=0.263)
	Exercise (m)	63.17±18.15	20.17±8.59 (p=0.082)	41.67±15.09 (p=0.441)
Moderate intensity activity	Cycling on flat ground	0.40±0.27	1.07±0.45 (p=0.395)	0.53±0.26 (p=0.476)
	Lift light weights	1.07±0.44	1.47±0.50 (p=0.548)	1.03±0.44 (p=0.992)
Walking	walk fast	1.90±0.52	1.70±0.54 (p=0.690)	2.23±0.55 (p=0.620)
	Walk slowly	5.00±0.53	4.33±0.59 (p=0.479)	3.87±0.57 (p=0.153)
	Walk at home	5.77±0.43	4.53±0.55 (p=0.286)	5.83±0.42 (p=0.302)
	Travel to various places	5.67±0.48	4.57±0.56 (p=0.151)	5.17±0.51 (p=0.602)
	Walk for relaxation	2.43±0.56	2.37±0.58 (p=0.862)	1.97±0.51 (p=0.678)
	Sit at the desk	2.67±0.57	2.37±0.59 (p=0.727)	2.23±0.54 (p=0.733)
Sitting	Sit and watch TV	3.10±0.57	4.73±0.57 (p=0.034)*	4.57±0.54 (p=0.048)*
	Sit and chat with friends	4.47±0.56	4.17±0.56 (p=0.751)	4.73±0.50 (p=0.937)
	Sit and relax	4.40±0.59	4.67±0.56 (p=0.609)	4.17±0.58 (p=0.829)
	Sit and read a book	0.93±0.33	1.53±0.50 (p=0.583)	1.83±0.52 (p=0.274)
Other activities		0.00 ± 0.00	0.00±0.00	0.00±0.00

*p-value < 0.05 compared to the placebo group

Table. Physical activity of adulthood volunteers who consumed placebo, or “Zuper rice” at the doses of 2, and 4 g per day at 6-week. (N=30/arm). Data are expressed as mean±S.E.M.

**Parameter**		**6-week**		
		Placebo (n=30)	Zuper rice 2 g/day (n=30)	Zuper rice 4 g/day (n=30)
high intensity activity	Run fast	0.00±0.00	0.37±0.37 (p=0.305)	0.09±0.09 (p=0.340)
	Lifting heavy objects	0.90±0.45	0.00±0.00 (p=0.043)*	0.27±0.27 (p=0.140)
	Exercise (m)	18.75±10.95	51.32±19.12 (p=0.264)	12.05±5.97 (p=0.840)
	Cycling on flat ground	0.60±0.42	0.68±0.47 (p=0.936)	0.64±0.39 (p=0.754)
Moderate intensity activity	Lift light weights	0.85±0.49	0.00±0.00 (p=0.083)	0.55±0.38 (p=0.560)
	walk fast	2.80±0.70	1.79±0.71 (p=0.152)	3.55±0.69 (p=0.523)
	Walk slowly	3.65±0.72	4.84±0.69 (p=0.336)	4.41±0.66 (p=0.589)
Walking	Walk at home	5.50±0.59	5.47±0.63 (p=0.901)	6.27±0.41 (p=0.494)
	Travel to various places	5.35±0.63	4.47±0.73 (p=0.539)	5.50±0.58 (p=0.833)
	Walk for relaxation	3.10±0.73	2.47±0.72 (p=0.470)	4.09±0.66 (p=0.441)
	Sit at the desk	1.95±0.62	3.11±0.74 (p=0.212)	2.68±0.65 (p=0.359)
	Sit and watch TV	3.35±0.70	5.05±0.65 (p=0.097)	4.59±0.64 (p=0.194)
Sitting	Sit and chat with friends	4.50±0.73	4.53±0.69 (p=0.938)	5.95±0.46 (p=0.216)
	Sit and relax	4.65±0.70	4.21±0.76 (p=0.615)	5.36±0.56 (p=0.602)
	Sit and read a book	0.40±0.35	1.84±0.70 (p=0.090)	2.09±0.60 (p=0.022)*
Other activities		0.00 ± 0.00	0.00±0.00	0.05±0.05 (p=0.305)

*p-value < 0.05 compared to the placebo group

Table. Physical activity of adulthood volunteers who consumed placebo, or "Zuper rice" at the doses of 2, and 4 g per day at 12-week (N=30/arm). Data are expressed as mean±S.E.M.

**Parameter**		**12-week**		
		Placebo (n=30)	Zuper rice 2 g/day (n=30)	Zuper rice 4 g/day (n=30)
high intensity activity	Run fast	0.00±0.00	0.00±0.00 (p=1.000)	0.19±0.13 (p=0.225)
	Lifting heavy objects	2.06±0.81	0.76±0.56 (p=0.247)	0.80±0.45 (p=0.151)
	Exercise (m)	55.33±19.82	46.15±22.17 (p=0.667)	35.95 ± 13.34 (p=0.422)
	Cycling on flat ground	0.73±0.48	0.53±0.53 (p=0.405)	0.19±0.19 (p=0.167)
Moderate intensity activity	Lift light weights	1.06±0.63	1.07±0.72 (p=0.809)	0.80±0.46 (p=0.889)
	walk fast	3.13±0.90	3.07±0.97 (p=0.959)	2.90±0.72 (p=0.872)
	Walk slowly	2.66±0.79	4.92±0.90 (p=0.062)	6.04±0.47 (p=0.001)***
Walking	Walk at home	4.93±0.63	4.84±0.93 (p=0.854)	6.33±0.39 (p=0.038)*
	Travel to various places	3.66±0.87	5.15±0.83 (p=0.319)	5.90±0.52 (p=0.041)*
	Walk for relaxation	2.06±0.81	2.92±0.95 (p=0.487)	4.00±0.72 (p=0.106)
	Sit at the desk	1.73±0.64	3.38±0.86 (p=0.187)	2.66±0.71 (p=0.670)
	Sit and watch TV	2.73±0.70	3.38±0.97 (p=0.576)	4.23±0.71 (p=0.125)
Sitting	Sit and chat with friends	4.20±0.69	4.69±0.91 (p=0.586)	5.47±0.50 (p=0.117)
	Sit and relax	4.26±0.74	5.53±0.78 (p=0.124)	5.66±0.55 (p=0.081)
	Sit and read a book	0.40±0.23	1.92±0.83 (p=0.193)	2.61±0.67 (p=0.033)*
Other activities		0.00 ± 0.00	0.00±0.00	0.00±0.00

*,**p-value < 0.05,0.001 compared to the placebo group
