# Supplementary material for: An Anthocyanin- and Anti-Ageing Amino Acids-Enriched Pigmented Rice Innovation Promotes Healthy Ageing Through the Modulation of Telomere, Oxidative Stress and Inflammation Reduction: A Randomized Clinical Trial
Source: Int J Mol Sci. 2025 Nov 11;26(22):10911. doi: 10.3390/ijms262210911 (PMC12652741; doi:10.3390/ijms262210911)
Supplement: Supplementary file 1 [file ijms-26-10911-s001.zip › Supplementary material file S5 FFQ ZUPER RICE.pdf]

Supplementary material file S5

Table. Food frequency questionnaire of volunteers who consumed placebo, or "Zuper rice " at the doses of 2 , and 4 g per day at baseline. (N=30/arm). Data are expressed as mean±S.E.M.

Parameters	Baseline		
	**Placebo** (*n*=30)	**Zuper rice 2 g/day** (*n*=30)	**Zuper rice 4 g/day** (*n*=30)
Rice cooked (scoop)	25.90±1.95	26.15±2.56 (p=0.783)	26.81±2.74 (p=0.988)
Purple rice cooked (scoop)	2.16±0.91	3.26±1.38 (p=0.697)	1.83±0.72 (p=0.725)
Meat (piece)	48.43±6.87	55.16±7.75 (p=0.673)	49.03±8.06 (p=0.706)
Egg	9.86±0.99	10.73±1.47 (p=0.899)	11.13±1.36 (p=0.486)
Milk (glass/box)	3.68±0.91	4.60±0.97 (p=0.427)	5.33±0.89 (p=0.086)
Vegetables (cup)	11.70±1.82	10.63±1.36 (p=1.000)	11.31±1.36 (p=0.917)
Fruit (cup)	10.45±1.82	8.31±1.24 (p=0.578)	9.56±1.35 (p=0.935)
Purple Vegetables or fruit (cup)	2.18±0.88	3.16±1.01 (p=0.300)	3.40±0.94 (p=0.083)

Table. Food frequency questionnaire of volunteers who consumed placebo, or " Zuper rice" at the doses of 2 , and 4 g per day at 6-week. (N=30/arm). Data are expressed as mean±S.E.M.

**Parameters**	**6-week**		
	**Placebo** (*n*=30)	**Zuper rice 2 g/day** (*n*=30)	**Zuper rice 4 g/day** (*n*=30)
Rice cooked (scoop)	27.12±2.83	24.92±2.13 (p=0.775)	29.13±2.78 (p=0.771)
Purple rice cooked (scoop)	3.25±1.22	4.10±1.87 (p=0.615)	1.22±0.60 (p=0.207)
Meat (piece)	37.70±5.28	52.94±9.03 (p=0.354)	52.13±11.20 (p=0.890)
Egg	9.25±1.34	11.42±1.80 (p=0.481)	12.18±1.93 (p=0.363)
Milk (glass/box)	4.85±1.21	6.36±1.52 (p=0.616)	7.90±1.30 (p=0.068)
Vegetables (cup)	15.22±2.74	10.44±1.68 (p=0.271)	13.63±1.71 (p=0.990)
Fruit (cup)	14.80±2.61	8.68±2.04 (p=0.063)	12.27±1.81 (p=0.622)
Purple Vegetables or fruit (cup)	4.95±1.45	4.07±1.53 (p=0.814)	3.34±1.10 (p=0.412)

Table. Food frequency questionnaire of volunteers who consumed placebo, or " Zuper rice" at the doses of 2 , and 4 g per day at 12-week. (N=30/arm). Data are expressed as mean±S.E.M.

**Parameters**	**12-week**		
	**Placebo** (*n*=30)	**Zuper rice 2 g/day** (*n*=30)	**Zuper rice 4 g/day** (*n*=30)
Rice cooked (scoop)	28.80±1.99	24.92±4.51 (p=0.433)	30.28±3.09 (p=0.741)
Purple rice cooked (scoop)	4.73±1.76	3.07±1.71 (p=0.394)	1.61±0.72 (p=0.158)
Meat (piece)	40.66±5.96	53.84±15.04 (p=0.908)	51.42±11.46 (p=0.974)
Egg	13.46±1.98	10.69±2.17 (p=0.381)	12.28±1.39 (p=0.822)
Milk (glass/box)	7.53±1.71	5.23±1.86 (p=0.164)	6.71±1.15 (p=0.723)
Vegetables (cup)	14.23±3.41	8.46±1.90 (p=0.221)	13.33±1.59 (p=0.531)
Fruit (cup)	15.83±3.27	7.76±2.24 (p=0.056)	11.26±2.05 (p=0.311)
Purple Vegetables or fruit (cup)	3.40±1.41	4.76±1.97 (p=0.697)	3.38±0.87 (p=0.611)
