# Supplementary material for: An Anthocyanin- and Anti-Ageing Amino Acids-Enriched Pigmented Rice Innovation Promotes Healthy Ageing Through the Modulation of Telomere, Oxidative Stress and Inflammation Reduction: A Randomized Clinical Trial
Source: Int J Mol Sci. 2025 Nov 11;26(22):10911. doi: 10.3390/ijms262210911 (PMC12652741; doi:10.3390/ijms262210911)
Supplement: Supplementary file 1 [file ijms-26-10911-s001.zip › Supplementary material file S6 Anthocyanin in various conditions.pdf]

Supplementary material file S6 Polyphenolic compounds, and anthocyanin content in "Zuper rice", Cooked Zuper rice", and Baked Zuper rice heated at 90°C for various perious ranging grom 5-30 minutes.

Sample	Total phenolic content (mg Gallic acid/g)	Total anthocyanin content (mg C3G /g)
Cooked Zuper rice (หุงสุก)	0.291±0.004	0.312±0.000
Zuper rice (ดิบ)	2.188±0.013	2.333±0.014
Baked Zuper rice at 90°C for 5 min.	2.078±0.011	2.416±0.013
Baked Zuper rice at 90°C for 10 min.	2.254±0.007	2.595±0.031
Baked Zuper rice at 90°C for 15 min.	1.984±0.009	2.143±0.035
Baked Zuper rice at 90°C for 20 min.	1.943±0.007	1.926±0.037
Baked Zuper rice at 90°C for 25 min.	2.102±0.008	2.046±0.021
Baked Zuper rice at 90°C for 30 min.	1.884±0.008	1.731±0.017
