# Supplementary material for: An Anthocyanin- and Anti-Ageing Amino Acids-Enriched Pigmented Rice Innovation Promotes Healthy Ageing Through the Modulation of Telomere, Oxidative Stress and Inflammation Reduction: A Randomized Clinical Trial
Source: Int J Mol Sci. 2025 Nov 11;26(22):10911. doi: 10.3390/ijms262210911 (PMC12652741; doi:10.3390/ijms262210911)
Supplement: Supplementary file 1 [file ijms-26-10911-s001.zip › Supplementary material file S7 SPIRIT 2025 editable checklist.pdf]

SPIRIT 2025 checklist of items to address in a randomized trial protocol*

Section / Topic	No	SPIRIT 2025 checklist item description	Reported on page no.
**Administrative information**			
Title and structured summary	1a	Title stating the trial design, population, and interventions, with identification as a protocol	page 1, in title
	1b	Structured summary of trial design and methods, including items from the World Health Organization Trial Registration Data Set	Page 1
Protocol version	2	Version date and identifier	17 October 2022
Roles and responsibilities	3a	Names, affiliations, and roles of protocol contributors	Khon Kaen Ethical Committee for Human Research, approved all experimental protocol used in this study in page 15.lines 437-438.
	3b	Name and contact information for the trial sponsor	PMU C, P 20, lines 670-671
	3c	Role of trial sponsor and funders in design, conduct, analysis, and reporting of trial; including any authority over these activities	Funder have no roles on the study, but Funder support and facilitate the competition of product in the market.
	3d	Composition, roles, and responsibilities of the coordinating site, steering committee, endpoint adjudication committee, data management team, and other individuals or groups overseeing the trial, if applicable	Institution Ethical Committee , and http://www.thaiclinical trials.org/show/TCTR20221017004 control and quality of design, study , Page 15, lines 436-444.
**Open science**			
Trial registration	4	Name of trial registry, identifying number (with URL), and date of registration. If not yet registered, name of intended registry	Date of registration12 October 2022, Thai Clinical Trial Registry in page 15, lines 438- 439. First posted date 17 October 2022.
Protocol and statistical analysis plan	5	Where the trial protocol and statistical analysis plan can be accessed	The information regarding the trial protocol was in pages 15-16 lines 435-491, the statistical analysis was in pages 19-20, lines 629-638
Data sharing	6	Where and how the individual de-identified participant data (including data dictionary), statistical code, and any other materials will be accessible	No data sharing, they can be provided on request, and this information was in Page 20, lines 679-680.
	7a	Sources of funding and other support (e.g., supply of drugs)	Page 20, lines 670-671.

Funding and conflicts of interest	7b	Financial and other conflicts of interest for principal investigators and steering committee members	No other conflict of interest, and this information was in page21, lines 685-686
Dissemination policy	8	Plans to communicate trial results to participants, healthcare professionals, the public, and other relevant groups (e.g., reporting in trial registry, plain language summary, publication)	Report the results in TCTR system, in IJMS, in Conferences.
**Introduction**			
Background and rationale	9a	Scientific background and rationale, including summary of relevant studies (published and unpublished) examining benefits and harms for each intervention	Scientific Background was in pages 1-3, lines 38-103, rationale information was in page 2, lines 61-64 and page 3, lines 92-98,
	9b	Explanation for choice of comparator	Page 2, lines 78-83.
Objectives	10	Specific objectives related to benefits and harms	Page 3, lines 103-109
**Methods: Patient and public involvement, trial design**			
Patient and public involvement	11	Details of, or plans for, patient or public involvement in the design, conduct, and reporting of the trial	This study involves healthy, aged 45-65 years, in page 16, lines 459-463.
Trial design	12	Description of trial design including type of trial (e.g., parallel group, crossover), allocation ratio, and framework (e.g., superiority, equivalence, non-inferiority, exploratory)	Type of trial was in page 15, lines 436-437, location was in lines 442-443, allocation ratio was in page 16, lines 471-472. Framework was in page 16, lines 478-491 and superiority was mentioned in page 3 lines 103-106.
**Methods: Participants, interventions, and outcomes**			
Trial setting	13	Settings (e.g., community, hospital) and locations (e.g., countries, sites) where the trial will be conducted	Setting at Faculty of Medicine, Khon Kaen University, Thailand and this was mentioned in page 15, lines 442-443.
Eligibility criteria	14a	Eligibility criteria for participants	This information was in page 16, lines 459-469.
	14b	If applicable, eligibility criteria for sites and for individuals who will deliver the interventions (e.g., surgeons, physiotherapists)	Physician and trained researchers. In page 16, lines 459-461.
Intervention and comparator	15a	Intervention and comparator with sufficient details to allow replication including how, when, and by whom they will be administered. If relevant, where additional materials describing the intervention and comparator (e.g., intervention manual) can be accessed	Pages 14- 15, lines 407- 432, supplementary material file S1, S7
	15b	Criteria for discontinuing or modifying allocated intervention/comparator for a trial participant (e.g., drug dose change in response to harms, participant request, or improving/worsening disease)	Page 16, Ines 490-491
	15c	Strategies to improve adherence to intervention/comparator protocols, if applicable, and any procedures for monitoring adherence (e.g., drug tablet return, sessions attended)	No strategy.

	15d	Concomitant care that is permitted or prohibited during the trial	This information was in the supplementary material regarding consent form in file S5.
Outcomes	16	Primary and secondary outcomes, including the specific measurement variable (e.g., systolic blood pressure), analysis metric (e.g., change from baseline, final value, time to event), method of aggregation (e.g., median, proportion), and time point for each outcome	Page 16, lines 487-490
Harms	17	How harms are defined and will be assessed (e.g., systematically, non-systematically)	In this study, the products were administered via oral route, the harms should be assessed systematically and if occur, an appropriate medical care should be applied, and this information was in supplementary material file S5.
Participant timeline	18	Time schedule of enrollment, interventions (including any run-ins and washouts), assessments, and visits for participants. A schematic diagram is highly recommended (see Figure)	This information was in file S5.
Sample size	19	How sample size was determined, including all assumptions supporting the sample size calculation	Page 15, lines 450-457.
Recruitment	20	Strategies for achieving adequate participant enrollment to reach target sample size	Page 15, line 446.
**Methods: Assignment of interventions**			
Randomization:			
Sequence generation	21a	Who will generate the random allocation sequence and the method used	Page 16, lines 471-473
	21b	Type of randomization (simple or restricted) and details of any factors for stratification. To reduce predictability of a random sequence, other details of any planned restriction (e.g., blocking) should be provided in a separate document that is unavailable to those who enroll participants or assign interventions	Page 16, lines 472-473
Allocation concealment mechanism	22	Mechanism used to implement the random allocation sequence (e.g., central computer/telephone; sequentially numbered, opaque, sealed containers), describing any steps to conceal the sequence until interventions are assigned	Page 16, line 472.
Implementation	23	Whether the personnel who will enroll and those who will assign participants to the interventions will have access to the random allocation sequence	No, Na this information was in page 16, lines 475-478.
Blinding	24a	Who will be blinded after assignment to interventions (e.g., participants, care providers, outcome assessors, data analysts)	Participants, outcome assessors, data analysts in page 16, lines 473-475.
	24b	If blinded, how blinding will be achieved and description of the similarity of interventions	To blind the participants, the identical products were administered, and for the outcome assessors, and data analysts were provided only code of subjects and

			the codes were hidden until the final analyse in an opaque envelop at PI and open after final analysis or in case of medical emergency occur. The mentioned information was in page 15,lines 431-432, and page 16, lines 475-478.
	24c	If blinded, circumstances under which unblinding is permissible, and procedure for revealing a participant's allocated intervention during the trial	Serious side effect occur and must provide appropriate medical care. Information was in supplementary material file S5..
**Methods: Data collection, management, and analysis**			
Data collection methods	25a	Plans for assessment and collection of trial data, including any related processes to promote data quality (e.g., duplicate measurements, training of assessors) and a description of trial instruments (e.g., questionnaires, laboratory tests) along with their reliability and validity, if known. Reference to where data collection forms can be accessed, if not in the protocol	Page 16, lines 480-493 and in supplementary material file S5.
	25b	Plans to promote participant retention and complete follow-up, including list of any outcome data to be collected for participants who discontinue or deviate from intervention protocols	List of measured outcomes were mentioned in page 16, lines 488-492.
Data management	26	Plans for data entry, coding, security, and storage, including any related processes to promote data quality (e.g., double data entry; range checks for data values). Reference to where details of data management procedures can be accessed, if not in the protocol	Data entry and analysis were performed during a concealment period, data analyst was blinded (page 16, lines 473-475). Data will be available on request page 681-682.
Statistical methods	27a	Statistical methods used to compare groups for primary and secondary outcomes, including harms	Pages19-20, lines 632-640.
	27b	Definition of who will be included in each analysis (e.g., all randomized participants), and in which group	All participants who participate in all visits. Page 19, line 633.
	27c	How missing data will be handled in the analysis	No missing data as shown in page 4, lines115-116.
	27d	Methods for any additional analyses (e.g., subgroup and sensitivity analyses)	No applicable.
**Methods: Monitoring**			
Data monitoring committee	28a	Composition of data monitoring committee (DMC); summary of its role and reporting structure; statement of whether it is independent from the sponsor and funder; conflicts of interest and reference to where further details about its charter can be found, if not in the protocol. Alternatively, an explanation of why a DMC is not needed	NO DMC. The report was submitted to PMU C and the experts were responsible for checking and approving the correctness and quality of report. Thy performed independently and were not influenced by sponsor and funder. In

			addition, we must report the results to the Ethical Committee to close the project so the report should be checked before approving the project to close.
	28b	Explanation of any interim analyses and stopping guidelines, including who will have access to these interim results and make the final decision to terminate the trial	Stopping guidelines involved the serious side effects, the decision will depend on the project physician who will explore the side effects and should contact Srinagarind Hospital, Faculty of Medicine to provide appropriate medical care if it is required. After report to the Ethic Committee, the project will be terminated if serious cases were observed in high amount and appeared to be associated with the intervention.
Trial monitoring	29	Frequency and procedures for monitoring trial conduct. If there is no monitoring, give explanation	It was performed by the on-site visits because the intervention is low risk so every 6 weeks is still in normal application which was reported to be around 4-6 weeks.
**Ethics**			
Research ethics approval	30	Plans for seeking research ethics committee/institutional review board approval	Khon Kaen Ethical Committee for Human Research has approved on 30 November 2022 (HE651411)
Protocol amendments	31	Plans for communicating important protocol modifications to relevant parties	P20, lines 434-442
Consent or assent	32a	Who will obtain informed consent or assent from potential trial participants or authorized proxies, and how	PI: Prof. Dr. Jintanaporn Wattanathorn, and co-ordinator; Assist Prof. Dr.Wipawee Thukham-mee
	32b	Additional consent provisions for collection and use of participant data and biological specimens in ancillary studies, if applicable	Data were collected at Research Institute for High Human Performance and Health Promotion, Faculty of Medicine, Kon Kaen University.
Confidentiality	33	How personal information about potential and enrolled participants will be collected, shared, and maintained in order to protect confidentiality before, during, and after the trial	All data were collected using unique IDs instead of names, and employing secure, encrypted tools. During a

			trial, data were shared using encrypted platforms and access controls, and stored in locked physical locations. After a trial, data is maintained through strict retention and destruction policies, and while stored, it's secured to prevent loss while maintaining confidentiality.
Ancillary and post-trial care	34	Provisions, if any, for ancillary and post-trial care, and for compensation to those who suffer harm from trial participation	Free medical management and reimbursement for expenses like travel or lost wages.

*We strongly recommend reading this checklist in conjunction with the SPIRIT 2025 Explanation and Elaboration and the SPIRIT 2025 Expanded Checklist for important clarifications on all the items. We also recommend reading relevant SPIRIT extensions. See www.consort-spirit.org

Citation: Chan A-W, Boutron I, Hopewell S, Moher D, Schulz KF, et al. SPIRIT 2025 statement: updated guideline for protocols of randomised trials. BMJ 2025;389:e081477. https://dx.doi.org/10.1136/bmj-2024-081477

© 2025 Chan A-W et al. This is an Open Access article distributed under the terms of the Creative Commons Attribution License (https://creativecommons.org/licenses/by/4.0/), which permits unrestricted use, distribution, and reproduction in any medium, provided the original work is properly cited.
