# Supplementary material for: An Anthocyanin- and Anti-Ageing Amino Acids-Enriched Pigmented Rice Innovation Promotes Healthy Ageing Through the Modulation of Telomere, Oxidative Stress and Inflammation Reduction: A Randomized Clinical Trial
Source: Int J Mol Sci. 2025 Nov 11;26(22):10911. doi: 10.3390/ijms262210911 (PMC12652741; doi:10.3390/ijms262210911)
Supplement: Supplementary file 1 [file ijms-26-10911-s001.zip › Supplementary material file S8 Nucleic acid sequence.pdf]

Supplementary material file S8: Nucleic sequence of Telomerase Reverse Transcriptase (TERT), and Telomerase RNA Component (TERC)

Telomerase Reverse Transcriptase (TERT): The protein component responsible for adding DNA sequence repeats to the telomere ends.

Gene Symbol: TERT

Gene ID: 7015

RefSeq Accession Number: NM_198253.3

UniProt Accession Number: O14746

GenBank Accession Number for Genomic Sequence: AY007685

NATURE.COM

Telomerase RNA Component (TERC): The RNA template that TERT uses to synthesize telomeric DNA repeats.

Gene Symbol: TERC

Gene ID: 7012

RefSeq Accession Number: NR_001566.1

GenBank Accession Number: U86046

ACADEMIC.OUP.COM
