# Supplementary material for: An Anthocyanin- and Anti-Ageing Amino Acids-Enriched Pigmented Rice Innovation Promotes Healthy Ageing Through the Modulation of Telomere, Oxidative Stress and Inflammation Reduction: A Randomized Clinical Trial
Source: Int J Mol Sci. 2025 Nov 11;26(22):10911. doi: 10.3390/ijms262210911 (PMC12652741; doi:10.3390/ijms262210911)
Supplement: Supplementary file 1 [file ijms-26-10911-s001.zip › Supplemntary material file S1 Amino acid profile Vitamin B.pdf]

Supplementary material S1 Amino acid profile, total protein, and vitamin B6, B9, B12 were reported by Central Laboratory (Thailand) Co., Ltd., Songkhla Branch, Thailand.

Amino acid profile	Zuper rice (g/100 g protein)	Method
Essential amino acid (EAA)		
Arginine	9.14	In–house method based on Association of Official Analytical Chemists (AOAC) Vol.72 No.6 (1989)
Histidine	2.63	
Isoleucine	4.00	
Leucine	7.09	
Lysine	3.43	
Methionine	1.94	
Phenylalanine	4.00	
Threonine	4.00	
Tryptophan	0.91	
Valine	5.94	
EAA	43.09	
Alanine	5.49	In–house method based on Association of Official Analytical Chemists (AOAC) Vol.72 No.6 (1989)
Aspartic acid	6.06	
Cystine	2.06	
Glutamic acid	13.49	
Glutamine	5.71	
Glycine	3.77	
Hydroxyproline	1.37	
Proline	5.03	
Serine	3.20	
Tyrosine	1.60	
NEAA	47.77	

Parameters	Zuper rice	Method
Dietary fiber (g/100 g)	10.8	AOAC (2019) 985.29
Vitamin B6 (mg/100 g)	0.014	In house method based on Analytica Chimica Acta569(2006) 169-175
Vitamin B9 (mg/100 g)	0.021	In house method based on Analytica Chimica Acta569(2006) 169-175
Vitamin B12 (mg/100 g)	Not detected	In house method based on Analytica Chimica Acta569(2006) 169-175
Fat (%)	3.49	Extraction of Crude Fat, AOAC 920

Amino acid profile	Zuper rice (g/100 g protein)	Method
Total Protein	8.75	In–house method TE-CH-042 based on AOAC (2019) 981.10

Vitamin B6, B9, B12 were reported by Central Laboratory (Thailand) Co., Ltd., Songkhla Branch, Thailand.
